# Comparative efficacy of seed biopriming and soil drenching with *Bacillus altitudinis* TM22 and *Bacillus atrophaeus* MCM61 on the suppression of Fusarium wilt of cotton

**DOI:** 10.1002/ps.70380

**Published:** 2025-11-17

**Authors:** Tahir Mahmood, Anam Moosa, Faisal Zulfiqar, Muhammad Naveed Aslam, Ohud Muslat Alharthy, Fatimah Hadadi, Ahmad F. Alhomodi, Seham Sater Alhelaify, Eman Fayad, Asim Alsenani, Salman Aloufi, Tolga İzgü

**Affiliations:** ^1^ Institute of Plant Protection, Chinese Academy of Agricultural Sciences Beijing China; ^2^ Department of Plant Pathology, Faculty of Agriculture and Environment The Islamia University of Bahawalpur Bahawalpur Pakistan; ^3^ Department of Horticultural Science, Faculty of Agriculture and Environment The Islamia University of Bahawalpur Bahawalpur Pakistan; ^4^ Korea University Seoul Republic of Korea; ^5^ Department of Biotechnology, College of Sciences Taif University Taif Saudi Arabia; ^6^ Department of Biology, Faculty of Science Al‐Baha University Al‐Baha Kingdom of Saudi Arabia; ^7^ Department of Biology College of Science and Arts, Najran University Najran Kingdom of Saudi Arabia; ^8^ Institute for BioEconomy (IBE), National Research Council (CNR) Florence Italy

**Keywords:** defense related enzymes, disease suppression, *Fusarium oxysporum* f. sp. *vasinfectum*, gene expression, growth promotion, seed biopriming, soil drenching

## Abstract

**BACKGROUND:**

*Bacillus* species suppress fungal pathogens by producing antimicrobial peptides. However, there is limited evidence of comparative effects of seed biopriming and soil drenching with *Bacillus* on pathogen suppression.

**RESULTS:**

Among six *Bacillus* species, *B. altitudinis* TM22 exhibited the highest *in vitro* inhibition of *Fusarium oxysporum* f. sp. *vasinfectum* (FOV) associated with *Fusarium* wilt of cotton. Lipopeptides (LPs) and polyketides (PKs) were extracted from the two most inhibitory strains: *B. altitudinis* TM22 and *B. atrophaeus* MCM61, where LPs from TM22 showed higher *in vitro* inhibition of FOV. Liquid chromatography‐time‐of‐flight mass spectrometry (LC‐TOF/MS) analysis detected iturin, fengycin, surfactin, bacillibactin, bacillomycin, bacilysin and bacillaene in TM22, whereas iturin was deficient in MCM61. TM22 produced larger amounts of hydrogen cyanide, correlating with its higher antagonistic potential. In a glasshouse experiment, TM22 and MCM61 applied via seed biopriming showed the lowest disease incidence and severity and improved plant biomass compared to soil drenching. Co‐application of TM22 and MCM61 improved chlorophyll a, b, carotenoids, photosynthesis rate, RWC, and stomatal conductance. Additionally, malondialdehyde, electrolyte leakage and hydrogen peroxide levels were lower in plants treated with TM22 + MCM61 + FOV. Moreover, the activity of polyphenol oxidase (PPO), peroxidase (POD), ascorbate peroxidase (APX), catalase (CAT), superoxide dismutase (SOD), β‐1,3‐glucanase (GLU), phenylalanine ammonia‐lyase (PAL) and chitinase (CHI) enzymes was the highest in TM22 + MCM61 + FOV‐treated plants. The expression of *HMGR, MPK3, GST, PAL, SOD, PPO, APX, POD* and *CAT* genes was the highest in plants subjected to co‐application of TM22 and MCM61 against FOV.

**CONCLUSION:**

Our study highlights the potential of *Bacillus* strains TM22 and MCM61 applied via seed biopriming and soil drenching in suppressing *Fusarium* wilt and enhancing biomass of cotton plants. © 2025 The Author(s). *Pest Management Science* published by John Wiley & Sons Ltd on behalf of Society of Chemical Industry.

## Graphical abstract

- Co‐application of *Bacillus* strains TM22 and MCM61 suppressed *Fusarium* wilt of cotton.
- Seed biopriming with *Bacillus* strains performed better than soil drenching.
- TM22 + MCM61 improved vegetative and physiological aspects in cotton plants.
- TM22 + MCM61 enhanced the activity of defense enzymes and defense gene expression.
- TM22 + MCM61 lowered disease induced oxidative stress markers.

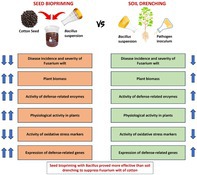

## 1 INTRODUCTION

*Fusarium oxysporum* f. sp. *vasinfectum* (FOV), associated with wilt disease in cotton (*Gossypium hirsutum* L.), is one of the most dangerous and devastating soil borne pathogens affecting yield and fiber quality.^1^ Several disease control strategies have been employed to mitigate the attack of *Fusarium* wilt of cotton, such as cultural practices, biological control, botanical extracts, resistant varieties and chemical pesticides.^2^ However, using chemical pesticides is highly undesirable because it is detrimental to human health and environmental safety. The use of chemical pesticides to fight against Fusarium wilt disease in cotton causes water and soil pollution, while disturbing beneficial microbes and environmental safety.^3^

Biological control of plant pathogens represents a promising alternative to chemical pesticides because they offer a sustainable, eco‐friendly management solution. Plant growth‐promoting rhizobacteria (PGPR) are one of the most promising biological control agents that protect the plants from pathogen attack and promote plant growth.^4^, ^5^ *Bacillus* species hold the most prominent position among all PGPR. *Bacillus* strains are regarded as ‘Generally Recognized As Safe’ (GRAS), according to the U.S. Food and Drug Administration, because they have no potential risks to the environment and ecosystem.^6^ They can form resilient endospores, which provide more consistent biocontrol activity under harsh environmental conditions compared to other PGPR, such as *Pseudomonas* species.^7^ *Bacillus* survives in various environmental conditions and soil types for extended periods by forming endospores, which tolerate harsh environments.^8^ The formation of endospores also improves the cell viability of *Bacillus* in commercial formulations. Several suppression mechanisms are utilized by *Bacillus* species, including the production of antimicrobial compounds such as lipopeptides (LPs) and polyketides (PKs), hydrolytic enzymes and volatile organic compounds, elicitation of induced systemic resistance (ISR), and competition for space and nutrients.^9^, ^10^ *Bacillus*‐derived LPs and PKs provide long‐lasting control without endangering human health or the environment, and they have promising antimicrobial efficacy.^11^ Iturin, surfactin, fengycin, bacilysin, bacillibactin, and several nonpeptides such as PKs, phospholipids and amino‐sugars, are among the most significant antimicrobial compounds that are produced by *Bacillus* species.^12^ The three prominent families of LPs–surfactin, iturin and fengycin–have exceptional antifungal properties; they improve the antifungal and antimicrobial activity of *Bacillus* species, facilitate the colonization of biological niches, decrease surface tension, inhibit spore germination, create ion channels in the fungal membranes, increase the permeability of the hyphal cells and plasma membrane, and promote the formation of biofilm.^10^

The search for novel *Bacillus* species with strong potential to suppress plant pathogens requires the detection of antimicrobial compounds produced by these *Bacillus* species.^13^ The techniques used for the detection of antimicrobial compounds produced by *Bacillus* species include: liquid chromatography time‐of‐flight mass spectrometry (LC‐TOF/MS), matrix‐assisted laser desorption/ionization time‐of‐flight mass spectrometry (MALDI‐TOF MS), gas chromatography–mass spectrometry (GC–MS) and high‐performance liquid chromatography (HPLC). Several researchers have used LC‐TOF/MS as one of the most reliable methods to detect antimicrobial compounds produced by *Bacillus* species because it is a highly sensitive and rapid detection method.^13^, ^14^

Plants activate their defense‐related enzymes such as superoxide dismutase (SOD), ascorbate peroxidase (APX), peroxidase (POD), catalase (CAT), polyphenol oxidase (PPO), phenylalanine ammonia‐lyase (PAL), β‐1,3‐glucanase (GLU) and chitinase (CHI) when exposed to LPs produced by *Bacillus* species. These defense‐related enzymes play vital roles in suppressing plant pathogens by activating a series of plant defense mechanisms.^15^ They support systemic acquired resistance (SAR) as an essential defense mechanism against plant pathogens.^16^ The LPs play an essential role in activating plant defense mechanisms, such as defense‐related gene expression, ISR and the production of antimicrobial compounds.^17^

In this study, we hypothesized that *Bacillus* species applied via seed priming and soil drenching can suppress Fusarium wilt of cotton by modulation of plant defense. Therefore, the present research aimed to assess the antagonistic potential of *Bacillus* species, particularly *B. altitudinis* TM22 and *B. atrophaeus* MCM61, against Fusarium wilt of cotton through a series of *in vitro* and glasshouse experiments. The study intended to detect the LPs and PKs produced by antagonistic *Bacillus* species through LC‐TOF/MS analysis. The study also intended to understand the mechanisms by which treatment with *Bacillus* affects defense‐related enzyme activities, defense‐related gene expression, physiological processes and stress markers in cotton plants.

## 2 MATERIAL AND METHODS

### 2.1 Fungal culture

Fungal culture was isolated from infected cotton plants, which showed typical symptoms of *Fusarium* wilt with visible vascular browning in the cross‐section of the stem. The cotton plant samples were collected from the Faqir Wali (62050), Haroon Abad, Bahawalnagar, Province Punjab, Pakistan (29.6081° N, 73.1468° E). The samples were excised into 5‐mm segments and surface‐disinfested with 2% sodium hypochlorite (NaOCl) and plated on potato dextrose agar (PDA) in 9‐cm Petri dishes and incubated at 25 ± 2 °C. The plates were observed every 12 h, and the fungal growth was transferred to fresh PDA medium (Oxoid Ltd, Hampshire, UK) immediately after it appeared. The identity of the fungal pathogen was verified based on morphological and molecular characterization. For morphological identification, the pathogen was observed at ×100 on an Olympus compound microscope (Olympus, Tokyo, Japan). The genomic DNA was extracted from the pathogen using PrepMan Ultra Reagent (Thermo Fisher Scientific, Waltham, MA, USA) at 100 °C for 15 min in a thermocycler (BioRad/ Thermo Fisher Scientific). The Internal Transcribed Spacer region (*ITS*) and Translation Elongation Factor (*TEF*) genes were amplified in a Thermocycler (Bio‐Rad, Hercules, CA, USA). The PCR reaction was carried out at the following conditions: initial denaturation for 5 min at 94 °C, 35 cycles of denaturation at 94 °C for 30 s, annealing for 30 s at 52 °C for *ITS*, 54 °C for *TEF*, followed by elongation for 30 s at 72 °C, with the final elongation performed at 72 °C for 8 min. The PCR product was purified and sent for sequencing to Macrogen (Seoul, Korea). The obtained nucleotide sequences of the pathogen were submitted to GenBank, NCBI and Isolation and identification of fungal culture. Phylogenetic analysis was performed to confirm the identity of the pathogen using mega (v11) software, and a maximum‐likelihood (ML) phylogenetic tree was made using the bootstrap method and Tamura–Nei model. The pathogenicity test was performed on healthy mature cotton plants to confirm Koch's postulates. Purified fungal culture was kept in an incubator (MIR‐154; Panasonic Healthcare Co. Ltd, Tokyo, Japan) at 26 ± 2 °C for further use in the subsequent experiments.

### 2.2 Bacterial cultures

The bacterial species were procured from the Molecular Plant Pathology Laboratory at the Islamia University of Bahawalpur, Punjab, Pakistan. Phylogenetic analysis was performed to confirm the identity of *Bacillus* species using mega (v11) software, and a neighbor‐joining phylogenetic tree was made using the bootstrap method and Tamura–Nei model. The bacterial species were preserved in a 30% glycerol stock solution at –80 °C. Before the experiments, the bacterial cultures were revived on Luria Bertani (LB) medium, and a 24‐h‐old culture was used for each experiment.

### 2.3 Dual culture assay

The antagonistic activity of six *Bacillus* species against the fungal pathogen was assessed in a dual culture plate assay *in vitro*. *Bacillus* species were grown in LB broth at 28 ± 1 °C for 24 h. FOV was cultured on PDA medium for 7 days at 25 ± 2 °C. A 5‐mm block of 7‐day‐old pathogen culture was placed at the center of a 90‐mm petri dish containing full‐strength PDA medium. Then, 5‐mm sterilized filter paper discs were placed at a 3 cm distance from the center on three sides of the Petri plate (one filter paper disc for control and two for treatments). Later, 5 μL of the respective *Bacillus* suspension was dropped on the sterilized filter paper discs using a micropipette, and the plates were incubated at 25 ± 2 °C for 5 days. At the control side, the filter paper discs were impregnated with 5 μL LB broth only. The zone of inhibition between the fungal colony (where the growth of the fungus was restricted) and the bacterial colony was measured 5 days postincubation (dpi). The inhibition zones were measured using a measuring scale in cm. Each *Bacillus* treatment had five replicates, and the *in vitro* assay was repeated thrice.^18^

### 2.4 Antifungal assay with lipopeptides

The two best‐performing *Bacillus* species from the antifungal assay (see Section 2.3) were selected to extract LPs. The strains were selected based on their strong antifungal activity and their consistent and reproducible results across multiple replicates and repeated experiments. The extraction of LPs from *Bacillus* species was carried out using the method described by Fatima *et al* ^14^ A 5‐mm culture block from a 7‐day‐old pathogen culture was inoculated at one side of full‐strength PDA medium, poured into a sterilized Petri dish (90 mm) and incubated at 25 ± 2 °C for 24 h. Then, 5 μL of 400 μg mL^−1^ LPs extract prepared in 0.01 m phosphate buffer solution (PBS) was poured on a sterilized filter paper disc placed on the other side of the plate. The concentration 400 μg mL^−1^ was selected based on our previous study.^19^ To assess the combined effect of TM22 and MCM61 the pathogen culture block was inoculated at the center of the plate and 5 μL of 400 μg mL^−1^ LPs extract was poured on sterilized filter paper discs placed on both sides of the plate. In the control group, only 5 μL PBS solution was poured on a filter paper disc. Inhibition was calculated in percentage 5 dpi. Inhibition was calculated using the following formula; Inhibition (%) = *C* – *T*/*C* × 100, where *C* is the growth of pathogen in control plate and *T* is the growth of pathogen in the treatment plate. Each treatment had five replicates, and the assay was performed twice under the same experimental settings.

### 2.5 Liquid chromatography time of flight mass spectrometry LC‐TOF/MS analysis

The LC‐TOF/MS analysis was carried out on a surveyor LC‐TOF/MS system (G2 QT of‐XS; Waters, Milford, MA, USA) using the protocol described by Hajji *et al*.^20^ to detect the LPs. A UPLC C18 2.1 × 100 mm column with ACQUITY UPLC BEH 1.7 μm particles was employed to perform the separations. The mobile phase to perform this analysis consisted of solution (A) formic acid (HCOOH) (0.1%) in water (H_2_O) and solution (B) HCOOH (0.1%) in acetonitrile (C₂H₃N). The methanolic samples were eluted in 5% solution A (formic acid 0.1% in H_2_O) for 2 min, followed by 15 min in 95% solution B (formic acid 0.1% in acetonitrile), and then 2 min in 95% solution B. The injection volume to conduct the analysis was 5 μL, and the flow rate was maintained at 200 μL min^−1^. The MS analysis was performed in the 50–1200 *m/z* range, with an electrospray source operating in positive ion mode and MSE acquisition mode. Following previously established parameters, LC‐TOF/MS modalities were applied to the chosen ions. The collision energy of 40 eV, 2.5 kV source voltage, 12 °C source temperature and 400 °C dissolution gas temperature was used for the LC‐TOF/MS analysis. masslynx (v 4.1) software was utilized to collect and process the obtained data from LC‐TOF/MS analysis.^17^

### 2.6 Hydrogen cyanide (HCN) production assay

The synthesis of HCN by *B. altitudinis* TM22 and *B. atrophaeus* MCM61 was tested in a qualitative assay.^21^ The specific King's B agar medium for testing HCN production was prepared by adding 20 g proteose peptone, 1.5 g magnesium sulfate heptahydrate, 1.5 g dipotassium hydrogen phosphate agar 20 g, and 1000 mL sterilized distilled water (SDW). The pH of the medium was maintained at 7.0. Briefly, 1 mL of 24‐h‐old culture of *B. altitudinis* TM22 and *B. atrophaeus* MCM61 was spread on King's B agar medium. Later, Whatman No. 1 filter paper (9 mm diameter) was soaked in 0.5% picric acid and 2% Na_2_CO_3_ solutions and placed in the plate's lid to detect the presence of HCN. The plates were tightly sealed with parafilm to trap the gas inside and prevent release. Later, these plates were incubated at 30 °C for 4 days. After 24–48 h, the color of the Whatman No. 1 filter paper changed from yellow to dark reddish‐brown, which was considered indicative of HCN production.^22^

### 2.7 In planta experiment

Cotton seeds cv. ‘SS32’ were surface‐disinfested with NaOCl solution (5%) for 1 min. The seeds were then rinsed thoroughly with SDW three to four times. The plants were given two different treatment applications: soil drenching and seed biopriming. For seed biopriming, 70 g cotton seeds were added to a container containing 250 mL of 24‐h‐old liquid *Bacillus* culture prepared in LB broth [1 × 10^8^ colony‐forming units (CFU) mL^−1^] [optical density at 600 nm (OD_600_) = 2.5] and suspended for 24 h. Later, the seeds were air‐dried and used for planting. The seeds were soaked in LB broth for 24 h for control treatment. The seeds were sown in clay loam soil filled in sterilized clean plastic pots with 14 cm height and a base diameter of 10 cm. The pots containing sterilized soil were inoculated with 50 mL of *Bacillus* culture prepared in LB broth (1 × 10^8^ CFU mL^−1^) (OD_600_ = 2.5) for soil drenching treatment. The healthy control plants were treated with 50 mL LB broth only and uninoculated with *Bacillus* or FOV suspensions. After 1 week, the treated seeds were sown in these pots (5 seeds per pot). The cotton plants were inoculated at four leaf stages with 1 × 10^5^ spore mL^−1^ conidial suspension of FOV. In a glasshouse, the pots were kept under controlled conditions at 25 °C temperature under a 16 h:8 h, light:dark photoperiod. Each treatment was repeated 10 times, and the experiment was performed thrice under the same experimental conditions. The treatments used in the experiment and how they were made is explained in Table 1. Disease assessments were made at 60 days post‐sowing (dps). Disease severity was measured using a 0–5 vascular browning scale.^23^ The vascular browning was observed by cutting the cross‐section of the stem vertically. On a 0–5 disease severity scale, 0 = no browning in the vascular system, 1 = 1–20% browning in the vascular system, 2 = 21–40% browning in the vascular system, 3 = 54–60% browning in the vascular system, 4 = 61–80% browning in the vascular system and 5 = 81–100% browning in the vascular system. Disease incidence for each treatment was calculated using the formula of Sukorini *et al* ^24^

**Table 1:** Treatments used in the glasshouse experiment

Sr. No.	Treatment	Application	
		Soil drenching	Seed biopriming
1	TM22 + FOV	Plants soil drenched with 50 mL of *B. altitudinis* TM22 (1 × 10^8^ CFU mL^−1^) (OD_600_ = 2.5) culture suspension prepared in LB broth and inoculated with a conidial suspension of FOV	Seeds soaked in 250 mL of 24‐h‐old *B. altitudinis* TM22 (1 × 10^8^ CFU mL^−1^) (OD_600_ = 2.5) suspension prepared in LB broth for 24 h
2	MCM61 + FOV	Plants soil drenched with 50 mL of *B. atrophaeus* MCM61 (1 × 10^8^ CFU mL^−1^) (OD_600_ = 2.5) culture suspension prepared in LB broth and inoculated with a conidial suspension of FOV	Seeds soaked in 250 mL of 24‐h‐old *B. atrophaeus* MCM61 (1 × 10^8^ CFU mL^−1^) (OD_600_ = 2.5) suspension prepared in LB broth for 24 h
3	TM22 + MCM61 + FOV	Plants soil drenched with co‐application of 25 mL *B. altitudinis* TM22 and 25 mL *B. atrophaeus* MCM61 (1 × 10^8^ CFU mL^−1^) (OD_600_ = 2.5) culture suspension prepared in LB broth and inoculated with a conidial suspension of FOV	Seeds soaked in 125 mL of 24‐h‐old *B. altitudinis* TM22 (1 × 10^8^ CFU mL^−1^) (OD_600_ = 2.5) for 24 h and then soaked for 24 h in 125 mL *B. atrophaeus* MCM61 (1 × 10^8^ CFU mL^−1^) (OD_600_ = 2.5) suspension prepared in LB broth
4	FOV	Plants treated with LB broth only and inoculated with conidial suspension of FOV only	Seeds soaked in 250 mL LB broth only for 24 h
5	HC	Healthy control plants treated with LB broth only and not inoculated with *Bacillus* or FOV suspensions	Seeds soaked in sterilized distilled H_2_O only for 24 h and not inoculated with *Bacillus* or FOV suspensions

TM22, *B. altitudinis* strain TM22; MCM61, *B. atrophaeus* strain MCM61; FOV, *F. oxysporum* f. sp. *vasinfectum*; HC, healthy control.

### 2.8 Assessment of vegetative traits

The leaf surface area, volume and length were measured at 60 dps on a Leaf Scanner (Perfection V800 Photo; EPSON, Amsterdam, Netherlands) with leaf scanning software (winfolia, model J221B). The root area, volume and length were measured on a root scanner (EPSON STD4800) using RhizoScanning software (winrhizo, model J221B).

### 2.9 Assessment of photosynthetic pigments

In order to assess photosynthetic pigments including chlorophyll a (Chl a), chlorophyll b (Chl b) and carotenoids, the extract of freshly harvested leaves was prepared in 80% acetone. The absorption was recorded at 663, 645 and 470 nm for Chl a, Chl b and carotenoid contents using a spectrophotometer (Shimadzu, Kyoto, Japan).^25^

### 2.10 Assessment of relative water content, rate of photosynthesis and stomatal conductance

In order to determine the relative water content (RWC) the third cotton leaf from the top was removed, and equal‐sized pieces of leaves were excised and their fresh weight (FW) was recorded on a digital weight measuring scale; the leaf pieces were soaked in SDW for 24 h so that the leaves may uptake H_2_O and become turgid then the turgid weight (TW) was recorded. Later, the leaf pieces were kept in an incubator for drying at 80 °C for 1 h and the dry weight (DW) was recorded. RWC was calculated by using the following formula: RWC (%) = (FW – DW)/(TW – DW) × 100.^26^ The rate of photosynthesis (Pn) in cotton plants, and stomatal conductance (gs) were measured on an infrared gas analyzer (IRGA) system (LI‐6400; Li‐Cor Inc., Lincoln, NB, USA) before harvest.^27^

### 2.11 Assessment of oxidative stress markers

The oxidative stress markers, including electrolyte leakage (EL), hydrogen peroxide (H_2_O_2_) and malondialdehyde (MDA) levels were measured in cotton plants. MDA level was assessed using the protocol of Cakmak & Horst^28^ with slight modification. The leaves were macerated in a clean pestle and mortar in 5 mL of 0.1% (w/v) trichloracetic acid (TCA). The resulting extract was centrifuged at 14 000 × *g* for 16 min at 4 °C. Then, 0.5 mL supernatant was collected, and 1.5 mL of 0.5% 2‐thiobarbituric acid (TBA; prepared in 20% TCA) was added in the supernatant and vortexed briefly, followed by incubation at 90 °C for 20 min. The reaction was terminated in an ice bath, and the sample was centrifuged at 14000 × *g* for 5 min at 25 °C. The absorbance of the reaction was measured at 600 nm wavelength on a spectrophotometer (Shimadzu, Japan).^29^ For the assessment of EL, four leaves per plant were taken from six randomly chosen cotton plants and excised into 1‐cm pieces. These pieces were rinsed four times with SDW to wash off the particles adhering to the surface and placed in separate vials containing 10 mL SDW. Then, these samples were incubated at room temperature (25 °C) on a shaking incubator (120 rpm) for 24 h.^30^ The reading for electrical conductivity of the bathing solution (EC1) was recorded postincubation, and the EC2 value was measured after autoclaving at 121 °C for 20 min; then, the solution was allowed to cool down for 30 min.^31^ The EL was calculated using the following formula; Electrolyte leakage (%) = EC1/EC2 × 100. To assess the H_2_O_2_ content, cotton leaf tissues of 0.2 g were macerated in liquid nitrogen in a clean pestle and mortar. Then, the macerated sample was homogenized in 2 mL reaction mixture containing 5 mm potassium phosphate buffer (pH 6.8) and 1 mm hydroxylamine. The reaction mixture was dissolved and centrifuged at 10 000 × *g* for 16 min at 4 °C. After centrifugation, the supernatant was collected, and 100 μL supernatant was added to 100 μm ferric ammonium sulfate, 25 mm of H_2_SO_4_, 250 μm of xylenol orange and 100 mm sorbitol, and the final volume was maintained at 2 mL. The mixture was incubated in completely dark conditions at 25 °C for 25–30 min. The absorbance of the mixture was measured in a spectrophotometer (Shimadzu) at 560 nm.^32^

### 2.12 Assessment of defense enzymes activities

The mature leaves of cotton plants from all treatments were taken to assess the activities of defense enzymes in 45‐day‐old cotton plants. To prepare the extract, the leaves were rinsed thoroughly, and 2 g cotton leaves were crushed in 0.1 m sodium phosphate buffer (10 mL) (pH 7.8) and centrifuged at 16 000 × *g* for 20 min at 2 °C. Later, the supernatant was collected and the pellet was thrown away. The enzyme activity was determined in the supernatant. The amount of total protein was determined by following the complete steps in Bradford's protocol.^33^ The activities of CAT, GLU, APX, SOD, CHI, POD and PPO were assessed following the protocol of Cao *et al* ^34^ The activity of PAL was measured using the protocol given by Ballester *et al* ^35^ The absorbance of every tested enzyme was measured at a specific wavelength for each enzyme on a spectrophotometer (Shimadzu, Japan). The activity of all enzymes was expressed in U mg^−1^ protein. The enzyme activity was assessed in five replicates of each treatment.

### 2.13 Gene expression analysis

The expression of defense‐related genes in cotton plants was measured in a quantitative real‐time polymerase chain reaction (qRT‐PCR) in a QuantStudio Real‐Time PCR machine (Thermo Fisher Scientific). RNA was extracted from cotton leaves using an RNA extraction kit (Omega Bio‐Tek, Norcross, GA, USA). To determine the extracted RNA's concentration and purity, a NanoDrop 1000 (Thermo Fisher Scientific), USA was used. An Evo M‐MLV reverse transcriptase (Accurate Biology, Hunan, China) kit was employed to prepare first‐strand cDNA. The expression of defense‐related genes–*HMGR*, *MPK3, GST, PAL, PPO, APX, POD, CAT* and *SOD*–were analyzed by qRT‐PCR with the SYBR Green Premix Taq HS qPCR kit (Accurate Biology). The primer information of the defense‐related genes and annealing temperatures are given in Table 2. Actin and beta‐tubulin were used as internal control genes to normalize the gene expression levels. Five replicates were tested from each treatment to determine the final expression levels of the target genes using the 2^−ΔΔCt^ method.^36^

**Table 2:** Primers used for expression analysis of defense‐related genes

No.	Gene	Code	Primers (5′ to 3′)	Annealing temperature (°C)	Function
01	Mitogen‐activated protein kinase 3	*MPK3‐F*	AAATACCCTAAGCCATCCACC	61	Signal transduction and gene activation
			CCAACCCAATTCCCATTTGTG		
		*MPK3‐R*			
02	3‐hydroxy‐3‐methylglutaryl‐coenzyme A reductase	*HMGR‐F*	GTTACAACCGAGGAAGACGAG	58	Activation of SAR, pathogen signaling
			CAATGGCAAACCCGATAACG		
		*HMGR‐R*			
03	Phenylalanine ammonia‐lyase	*PAL‐F*	ATGTTTGCTCAGTTTTCGGAAC	59	Antimicrobial compound, involved in lignin synthesis
		*PAL‐R*	GGCACTTTGAACATGGTTGG		
04	Glutathione‐S‐transferase	*GST‐F*	TCAGTGCTTTCCTACCCTTTG	60	ROS detoxification, Protection against oxidative damage
		*GST‐R*	ATACCCAACAGAGCTAGCAAC		
05	Superoxide dismutase	*SOD‐F*	CTGCCTCTGTCTCGATCATTG	58	Activation of antioxidant defense
		*SOD‐R*	ACCTTTCTGAATAGCCTCATGG		
06	Polyphenol oxidase	*PPO‐F*	GAGTCAAGGTTCGTGATAGCC	59	Antifungal compound, involved in cell death
		*PPO‐R*	GGTGATGTTCTTTGTTTCGGC		
07	Ascorbate peroxidase	*APX‐F*	TTCCAAAGGGCTAACACACATC	60	ROS scavenging, H_2_O_2_ detoxification
		*APX‐R*	CGAAGCATGATAGGAGCGCA		
08	Catalase	*CAT‐F*	TGGACCCAGAGGTCCGATT	60	Control the level of ROS, H_2_O_2_ detoxification
		*CAT‐R*	TCCAGGTGCTCGCAGAAAAT		
09	Peroxidase	*POD‐F*	CAGTGTCGGCAGAACTCACT	58	Strengthens cell wall, ROS modulation, and involved in plant defense
		*POD‐R*	GGTGTCCCACGATCGTTTCT		
10	Actin	*ACT‐F*	TTAGCCCCAAGCAGCATGAA	60	Involved in cell shape, movement and cell division
		*ACT‐R*	TGAGAACGCCTCTGTTTGTAAG		
11	Beta‐tubulin	*B‐TUB1‐F*	TGTGCCATGTATTGTGGCAA	59	Involved in intracellular transport and cell structure
			GTGTCTATTGCCTGGGGCAT		
		*B‐TUB1‐R*			

### 2.14 Statistics analysis

Analysis of all the data obtained in this study was conducted using the Spss statistical package (v29). All of the experiments were performed in a completely randomized design (CRD) under controlled conditions. The comparisons in treatment means were made using post hoc Tukey's honestly significant difference (HSD) at a significance level of *P* ≤ 0.05 after appropriate ANOVA.

## 3 RESULTS

### 3.1 Identification of fungal pathogen

**Figure 1:**
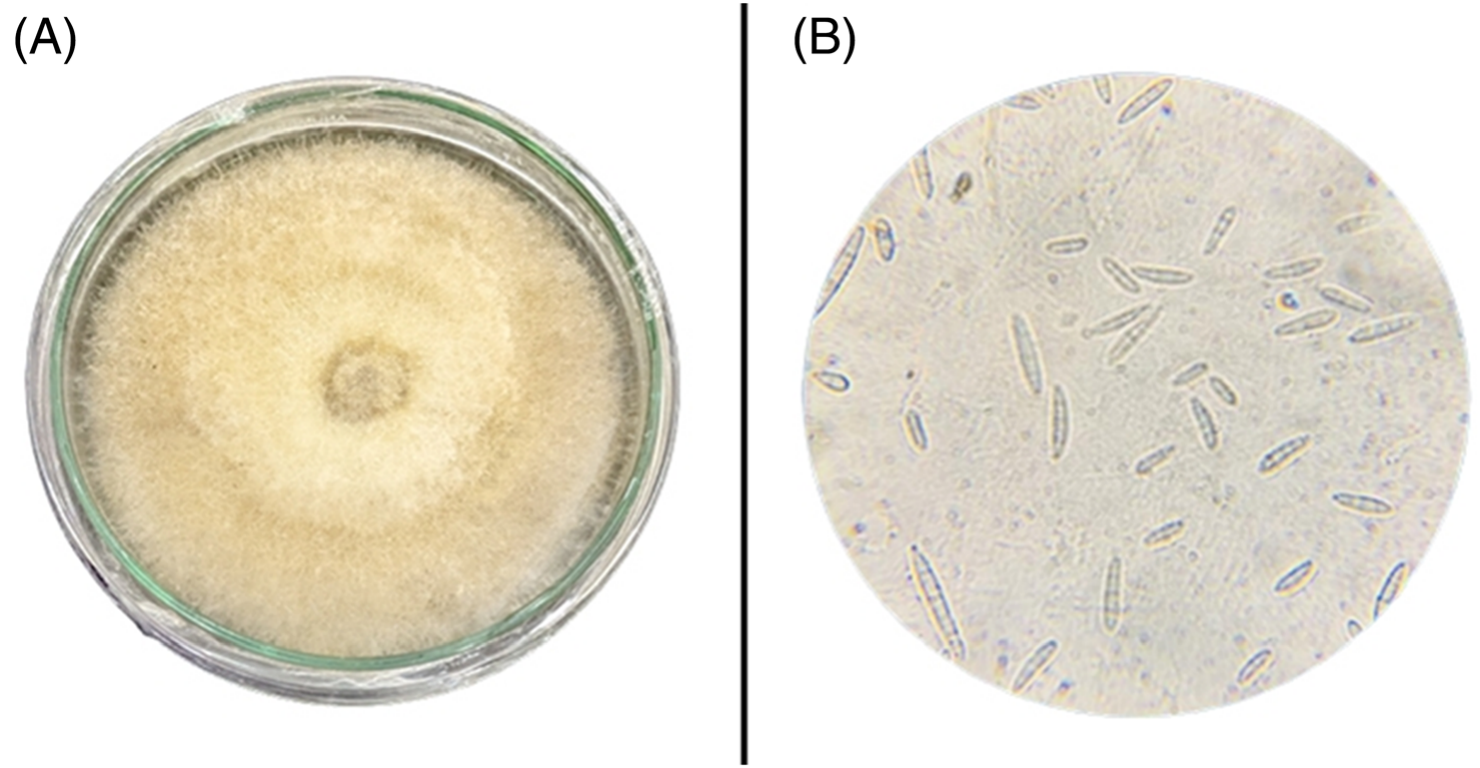
Colony morphology and microscopic features of *F. oxysporum* f. sp. *vasinfectum*: (A) pure culture plate of FOV on PDA; (B) microscopic view of conidia at ×100 magnification.

The pathogen developed dense, white, cottony colonies on PDA [Fig. 1(A)]. Under the microscope, both macroconidia and microconidia were observed at 100× [Fig. 1(B)]. Macroconidia were slightly curved or straight with three to five septations with foot‐shaped basal cell measuring 19.1 to 38.5 × 3.5 to 5.3 μm (*n* = 40). Microconidia were also observed. They were oval‐shaped without any septa and appeared on short monophialides measuring 5.8–11.2 to 2.7–4.9 μm (*n* = 40) [Fig. 1(B)]. The sequence obtained after molecular sequencing of *ITS* and *TEF* genes was submitted to GenBank, NCBI with ITS accession no. OR523451.1 and TEF accession no. PV324757.1 as *F. oxysporum* TM23. Phylogenetic analysis of TM23 showed 100% identity with *F. oxysporum* type strain NRRL25387 based on a ML tree (Supporting Information, Fig. S1).

### 3.2 Identification of bacterial cultures

Six different *Bacillus* strains were identified in this study based on molecular identification and phylogenetic analysis (Table 3). Based on phylogenetic analysis there were six different clades and *B. mojavensis* was used as an outgroup. The strain CFGP92 grouped together with *B. amyloliquefaciens* accession nos OP888971, FN652780 and KY777246. Strain RB58 appeared in a separate clade with *B. velezensis* OQ920283 and OQ626815. MCM61 grouped together with *B. atrophaeus* accession no. AF272016, MW879354 and MT254069. The strains S2 and MGRP21 were placed in a single clade with *B. subtilis* accession nos JX977127 and MW401274. In the fifth clade grouped together with two isolates of *B. altitudinis* accession nos OQ408169 and MK608736 (Fig. S2).

**Table 3:** Information about bacterial (*Bacillus*) species used in this study

Sr. No.	Species	Species code	Accession no.	
			*16S rRNA*	*gyrA*
1	*B. atrophaeus*	MCM61	OR660387	PV366746
2	*B. velezensis*	RB58	PV382507	PV271398
3	*B. subtilis*	MGRP21	OR660386	PV366747
4	*B*. a*myloliquefaciens*	CFGP92	OR660381	PV366745
5	*B. subtilis*	S2	OR660388	PV366748
6	*B. altitudinis*	TM22	OR660389	PV366749

### 3.3 *In vitro* antagonism assay

**Figure 2:**
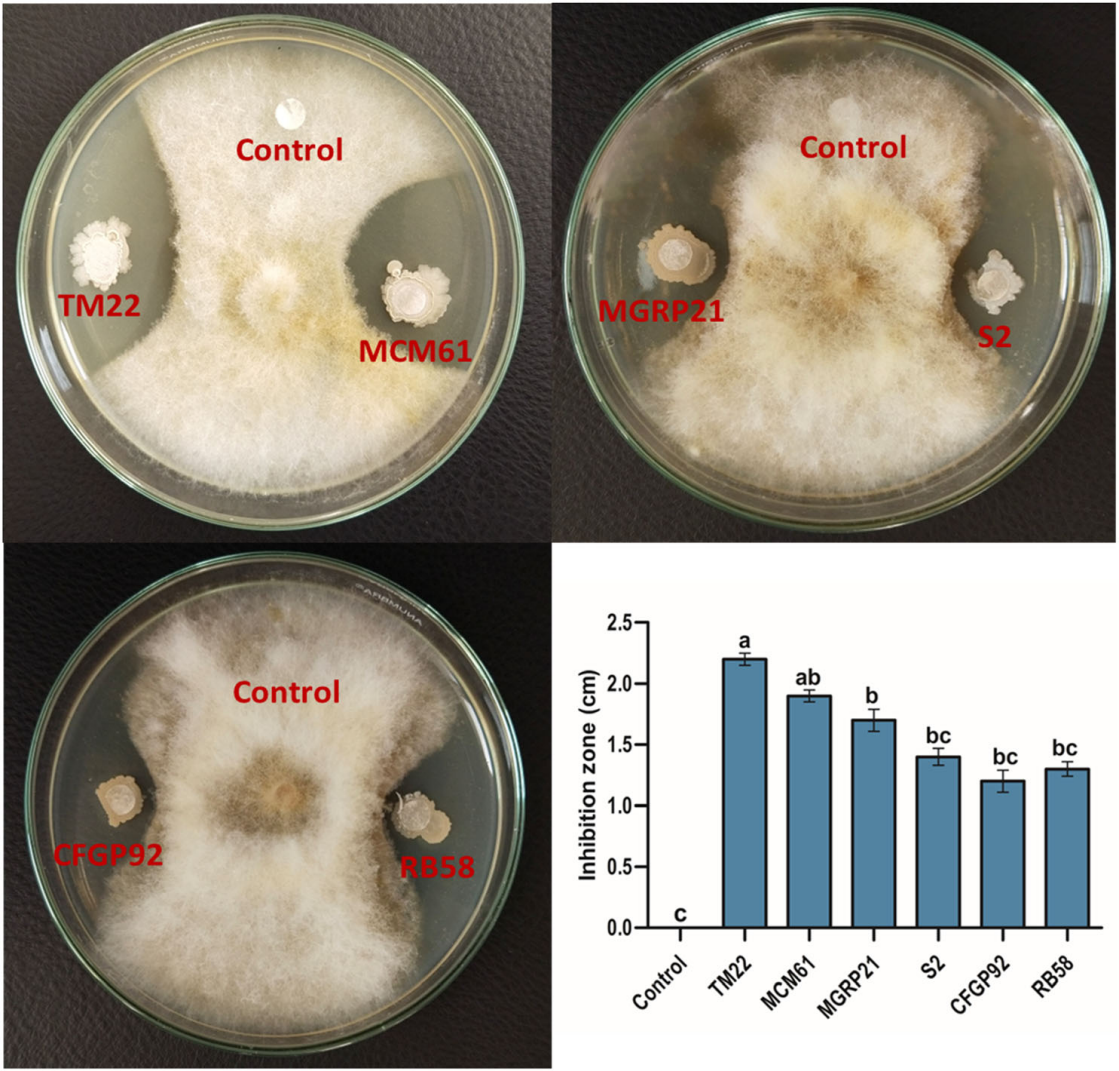
*In vitro* antagonism of *F. oxysporum* f. sp. *vasinfectum* by *Bacillus* species in a dual culture assay. The comparisons among the treatments were made by applying post hoc Tukey's HSD test, with small letters placed over the column indicating different treatment groups based on significant differences at *P* ≤ 0.05.

In an *in vitro* antagonism assay, all tested *Bacillus* species exhibited considerable inhibition of the colony growth of FOV. *B. altitudinis* TM22 caused the highest inhibition compared to other treatments (Fig. 2). TM22 produced an inhibition zone of 2.2 cm. *B. atrophaeus* MCM61 was the second most inhibitory strain, producing an inhibition zone of 1.9 cm. *B. amyloliquefaciens* CFGP92 produced the lowest inhibition with an inhibition zone of 1.2 cm (Fig. 2).

### 3.4 Antagonism assay with lipopeptides

**Figure 3:**
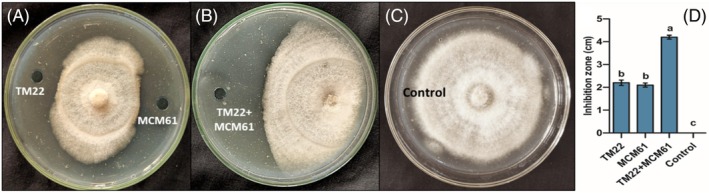
Antifungal efficacy of lipopeptides against colony growth of the pathogen: (A) Treatment with *B. altitudinis* TM22 at left side of the plate and treatment with MCM61 at right side of the plate; (B) combined treatment with *B. altitudinis* TM22 and *B. atrophaeus* MCM61; (C) control treatment (uninoculated with *Bacillus*); and (D) measurement of inhibition zones produced by *Bacillus* species. The comparisons among the treatments were made by applying post hoc Tukey's HSD test, with small letters placed over the column indicating different treatment groups based on significant differences at *P* ≤ 0.05.

LPs were extracted from *B. altitudinis* TM22, and *B. atrophaeus* MCM61 produced considerable inhibition of the colony growth of FOV. The clear zones around the fungal colonies indicated the inhibition of the pathogen by the respective *Bacillus* species applied as single treatments [Fig. 3(A)]. In the case of combined treatment where both TM22 and MCM61 were applied together considerably highest inhibition was produced compared to single treatments [Fig. 3(B)] and the control plate had no inhibition zones [Fig. 3(C)]. TM22 produced the higher inhibition of the colony growth of FOV with an inhibition zone of 2.4 cm compared to MCM61 where the inhibition zone was 1.5 cm [Fig. 3(D)].

### 3.5 Liquid chromatography time of flight mass spectrometry LC‐TOF/MS analysis

**Figure 4:**
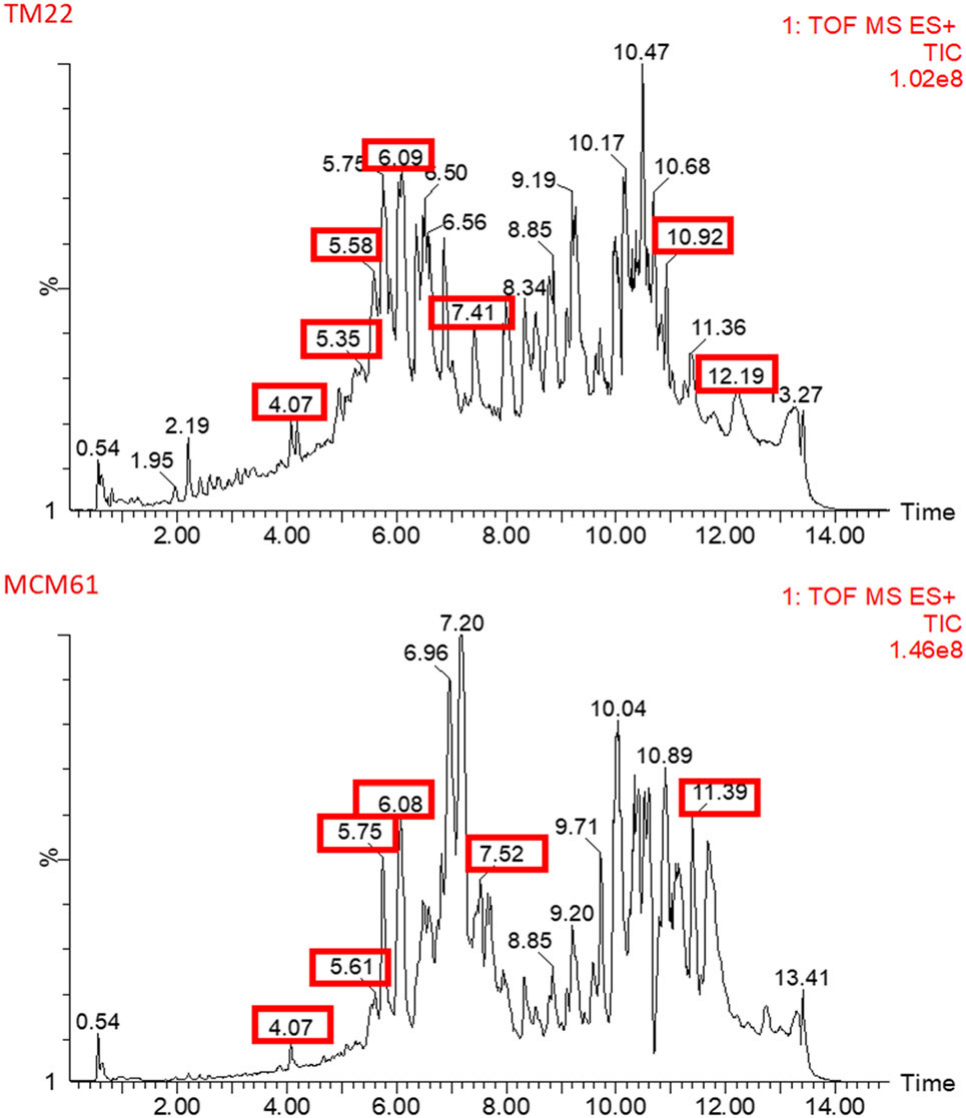
Liquid chromatography time of flight mass spectrometry analysis based on retention time for *B. altitudinis* TM22 and *B. atrophaeus* MCM61.

**Figure 5:**
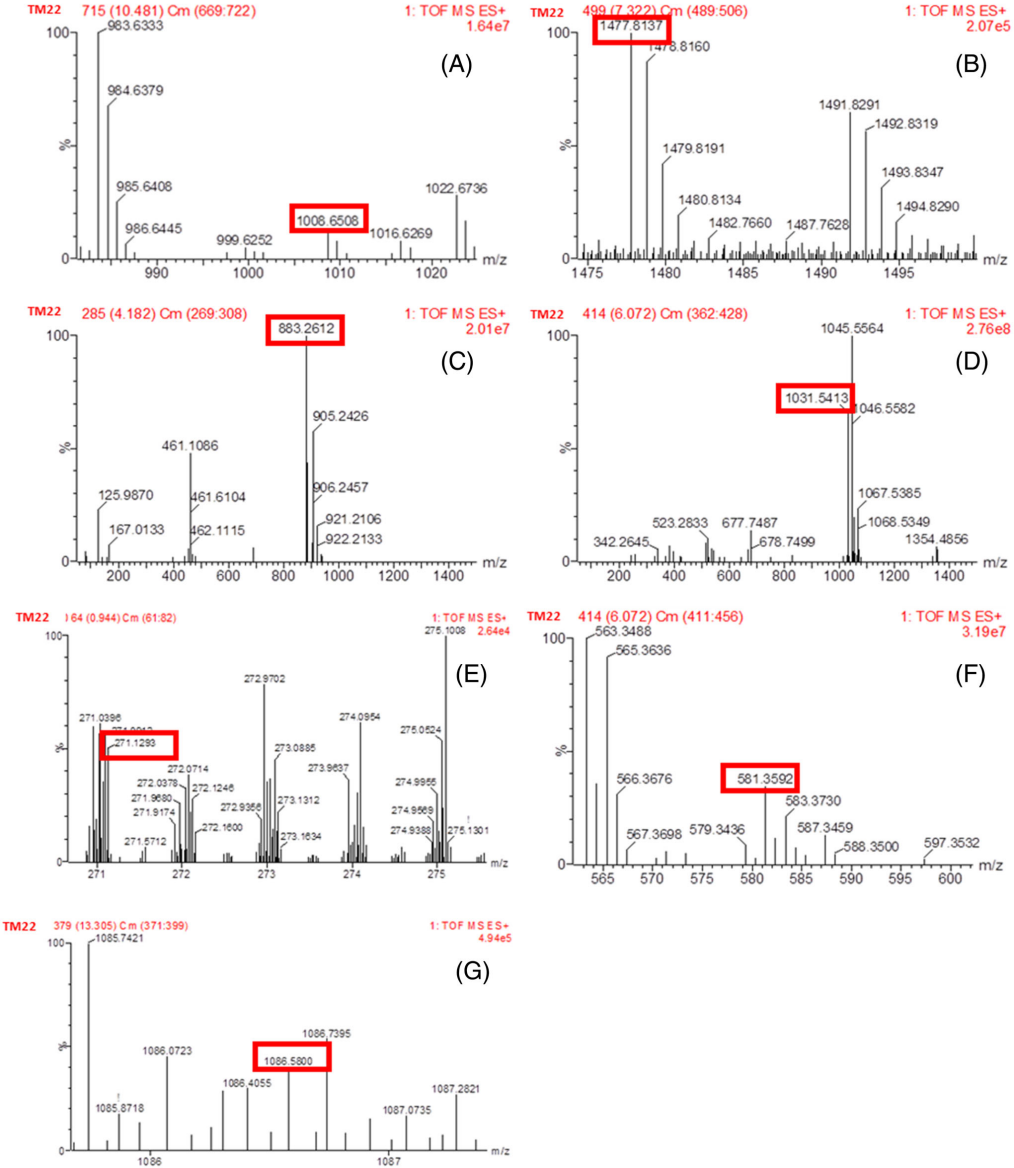
Liquid chromatography time of flight mass spectrometry analysis of *B*. *altitudinis* TM22: peaks for (A) surfactin, (B) fengycin, (C) bacillibactin, (D) bacillomysin, (E) bacilysin; (F) bacillaene and (G) iturin.

**Figure 6:**
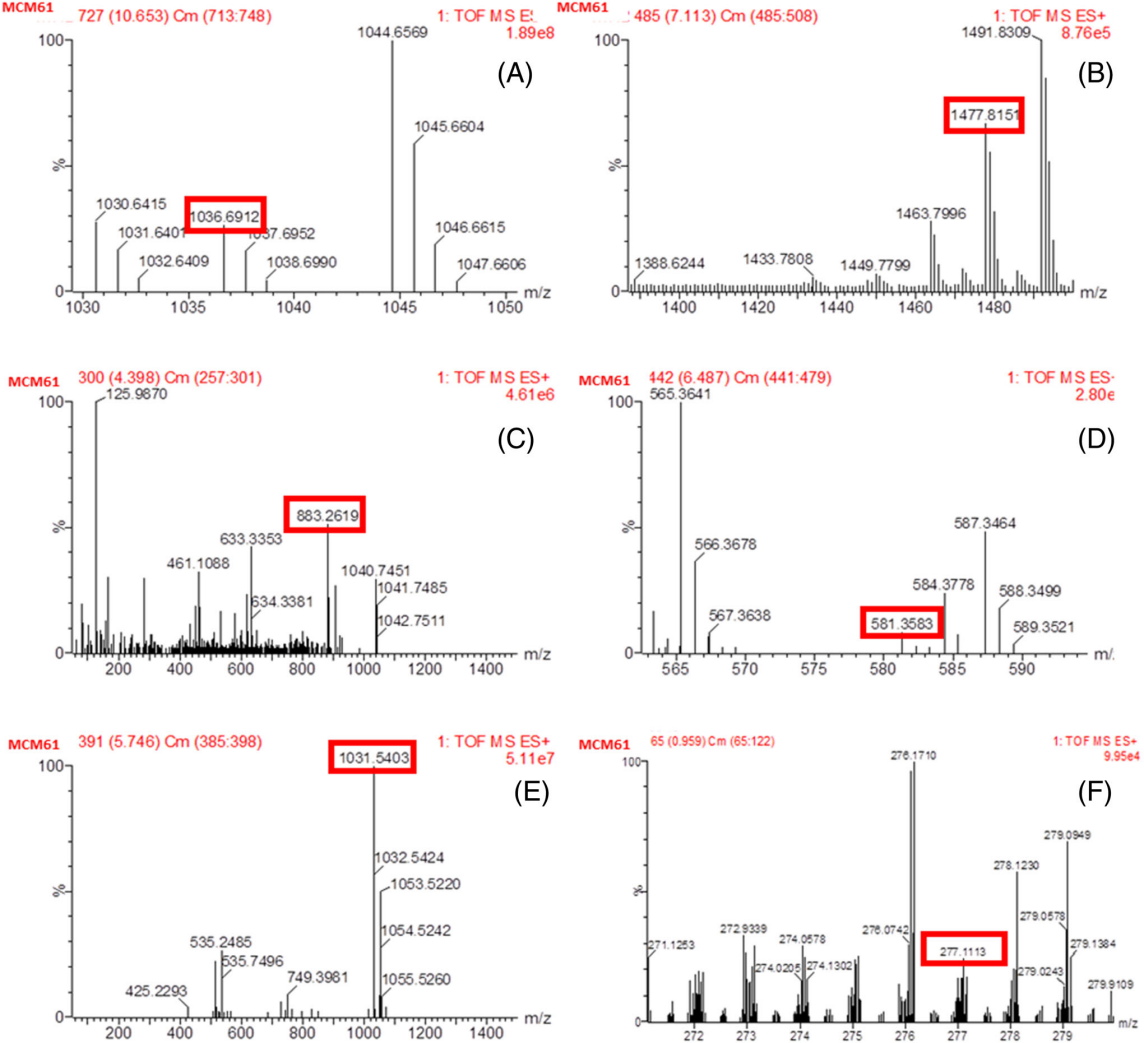
Liquid chromatography time of flight mass spectrometry analysis of *B. atrophaeus* MCM61: peaks for (A) surfactin, (B) fengycin, (C) bacillibactin, (D) bacillaene, (E) bacillomysin and (F) bacilysin.

LC‐TOF/MS analysis detected the presence of six LPs–surfactin, fengycin, iturin, bacilysin, bacillibactin and bacillomysin– and one PK bacillaene in *B*. *altitudinis* TM22 at different retention times (RT). The LPs were separated based on *m/z* values by comparing these values with reported peaks in previous literature and their corresponding retention times are reported in Table 4. Surfactin, fengycin A, iturin, bacilysin, bacillibactin, bacillaene and bacillomysin were detected at different peaks of *m/z* 1008.65 (10.92 min RT), 1447.81 (7.41 min RT), 1086.58 (12.19 min RT), 271.12 (4.07 min RT), 883.26 (5.35 min RT), 581.35 (6.09 min RT) and 1031.54 (5.58 min RT), respectively (Figs 4 and 5) (Table 4). The LC‐TOF/MS analysis of *B. atrophaeus* MCM61 revealed the presence of five LPs (surfactin, fengycin, bacilysin, bacillibactin, bacillomysin) and one PK bacillaene. By comparing the peaks with previous literature, the detected LPs and PK were identified based on m/z values and RT. Surfactin, fengycin, bacilysin, bacillibactin, bacillaene and bacillomysin were detected at peaks of *m/z* 1036.69 (11.39 min RT), 1477.81 (7.52 min RT), 271.121 (4.07 min RT), 883.26 (5.61 min RT), 581.35 (6.08 min RT) and 1031.54 (5.75 min RT), respectively (Figs 4 and 6) (Table 4).

**Table 4:** Assignment of the fragments obtained from *B. altitudinis* TM22 and *Bacillus atrophaeus* MCM61 to corresponding lipopeptides or polyketides by LC‐TOF/MS analysis based on retention time and mass‐to‐charge ratio

Strain	Peak	Mass M/S [M + H]^+^	Lipopeptide family	Retention time (min)	Reference
TM22	1	1008.65	Surfactin	10.92	37
	2	1477.81	Fengycin A	7.41	37
	3	883.26	Bacillibactin	5.35	38
	4	271.12	Bacilysin	4.07	39
	5	1031.54	Bacillomycin D	5.58	37
	6	581.36	Bacillaene	6.09	40
	7	1086.58	Iturin	12.19	41
MCM61	1	1036.69	Surfactin	11.39	37
	2	1477.81	Fengycin A	7.52	37
	3	883.26	Bacillibactin	5.61	38
	4	581.36	Bacillaene	6.08r	40
	5	1031.54	Bacillomycin D	5.75	37
	6	271.12	Bacilysin	4.07	39

### 3.6 Hydrogen cyanide (HCN) production assay

**Figure 7:**
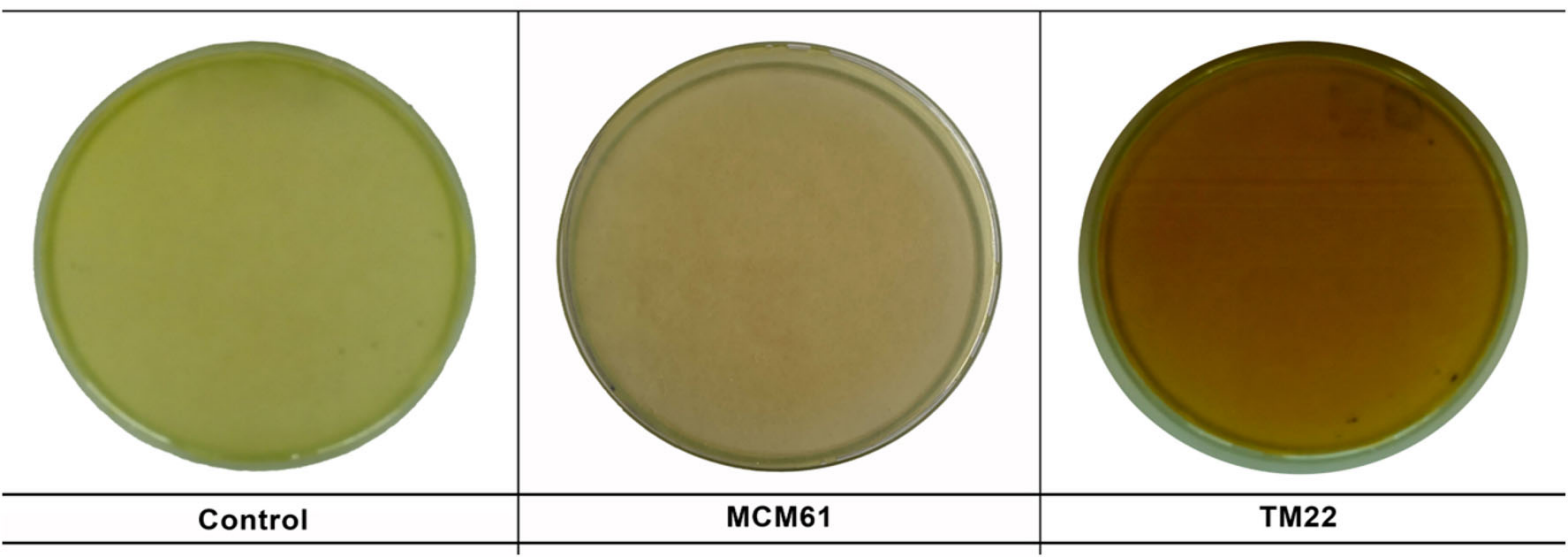
Hydrogen cyanide‐producing ability of *Bacillus* species on King's B agar medium.

The change in color from light yellow to light reddish‐brown and dark reddish‐brown confirms that HCN is produced by *B. atrophaeus* MCM61 (Fig. 7) and *B. altitudinis* TM22 (Fig. 7). *B. altitudinis* TM22 showed a darker color, indicating higher HCN production, which corresponds to the more substantial antagonism potential of TM22. In the control plate, the color of the medium was light yellow (Fig. 7).

### 3.7 Greenhouse experiment

**Figure 8:**
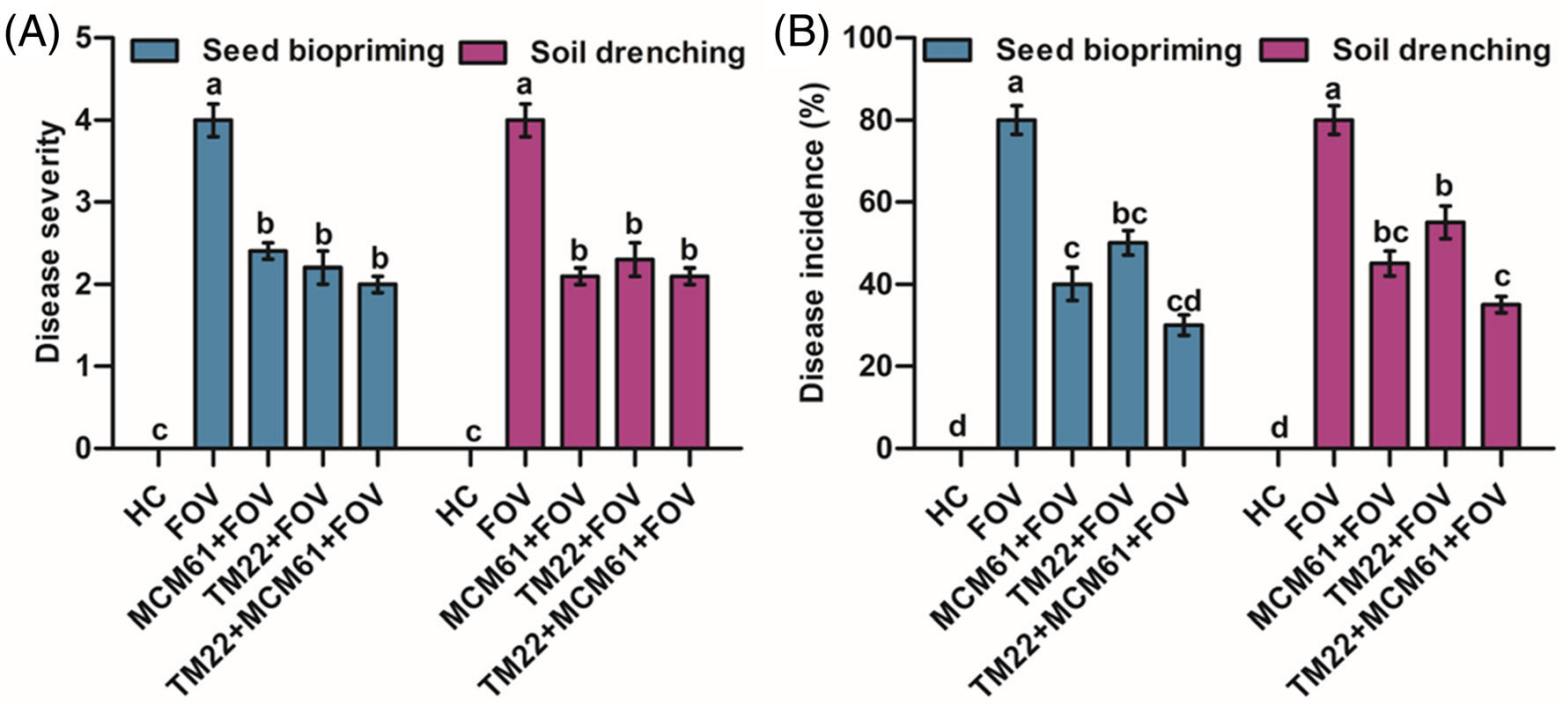
Disease assessment of *Fusarium* wilt of cotton in the glasshouse experiment: (A) disease severity and (B) disease incidence. The treatments were compared by applying post hoc Tukey's HSD test, with small letters placed over the column indicating different treatment groups based on significant differences at *P* ≤ 0.05.

The co‐application of *B. altitudinis* TM22 and *B. atrophaeus* MCM61 applied via seed biopriming showed the lowest disease severity 2.0 [Fig. 8(A)] and disease incidence of 30% [Fig. 8(B)] of *Fusarium* wilt of cotton as compared to the infected control, whereas in the case of soil drenching, combined treatment showed the lowest disease severity 2.1 [Fig. 8(A)] and disease incidence 35% [Fig. 8(B)] indicating the combined or additive effect of *Bacillus* species in disease suppression. In infected control plants, the disease severity was 4.0, and the incidence was 80%. Healthy control plants were asymptomatic and disease free.

### 3.8 Vegetative traits

**Figure 9:**
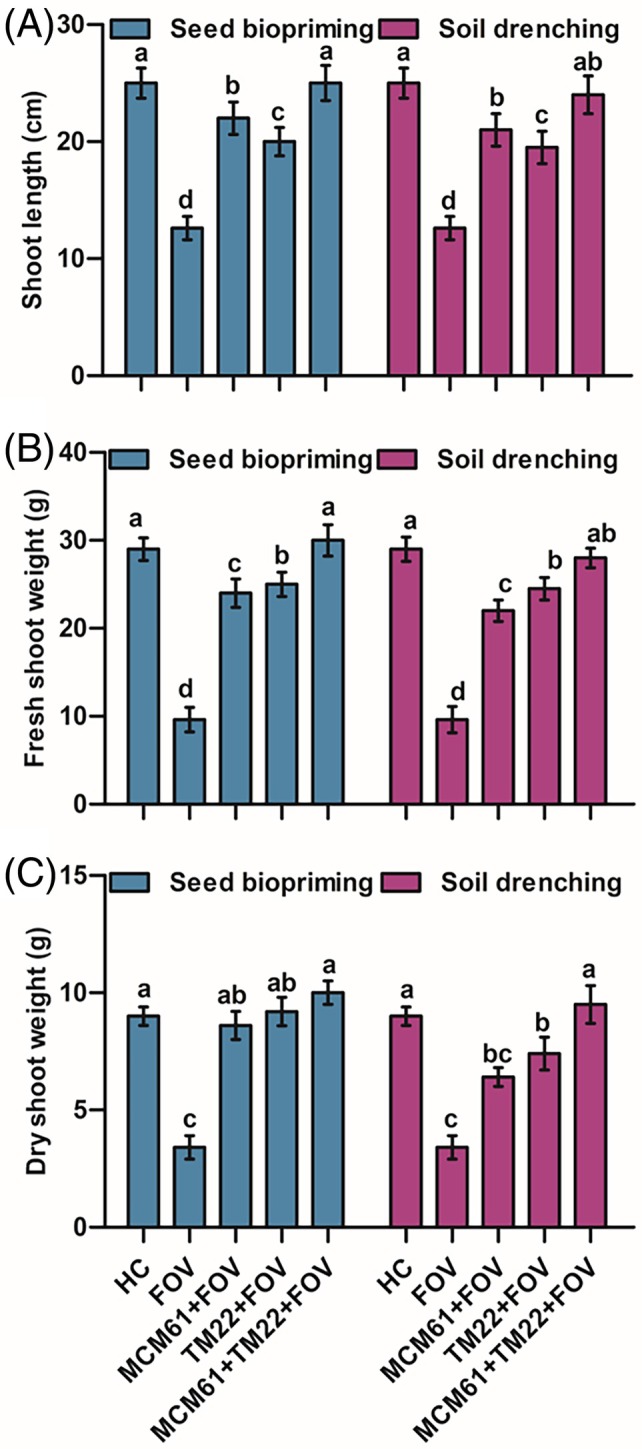
Effect of *Bacillus* species on vegetative traits of plants applied via seed biopriming and soil drenching: (A) shoot length, (B) shoot FW and (C) shoot DW. The comparisons among the treatments were made by applying post hoc Tukey's HSD test, with small letters placed over the column indicating different treatment groups based on significant differences at *P* ≤ 0.05.

**Figure 10:**
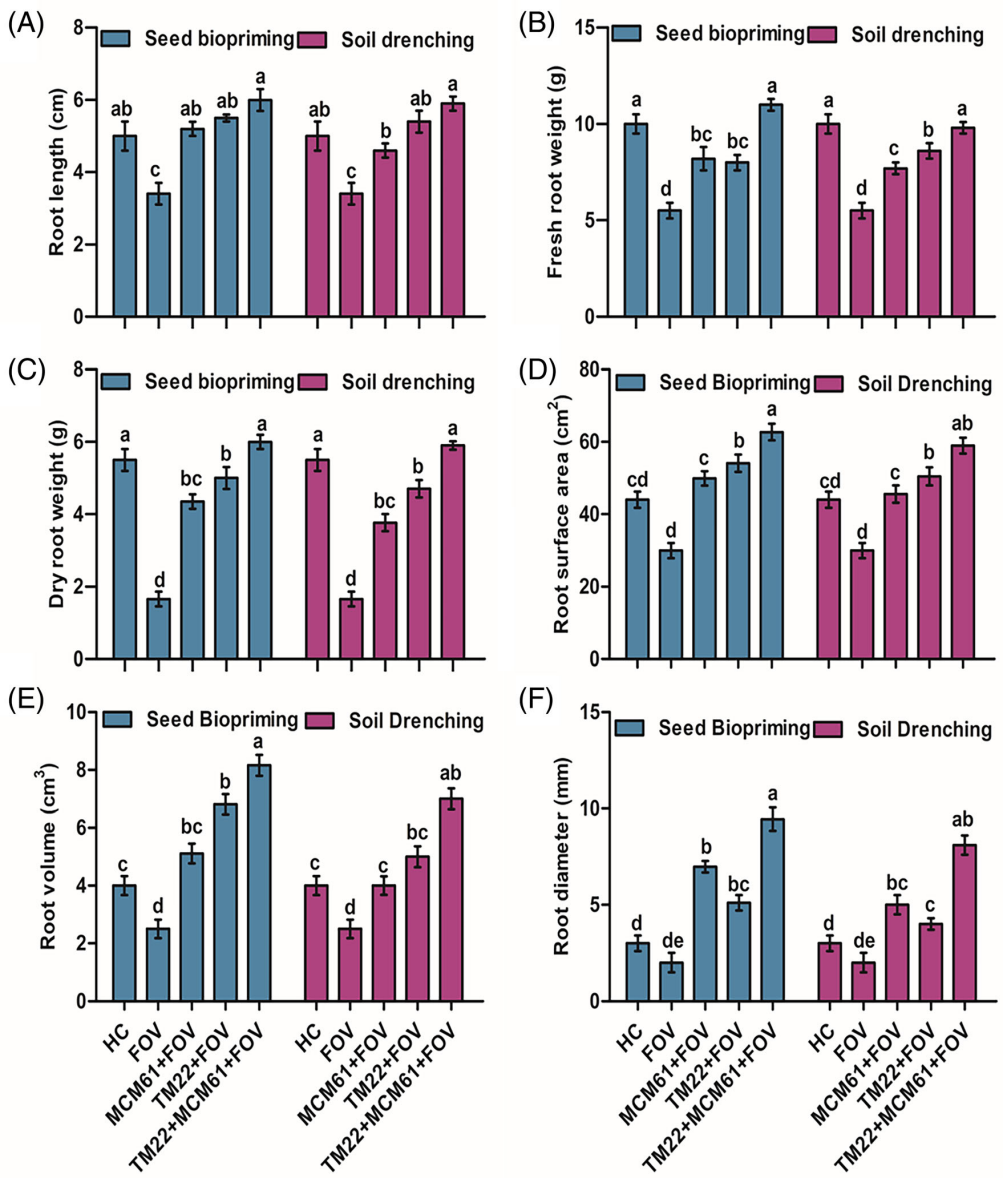
Effect of *Bacillus* species applied via seed biopriming and soil drenching on root growth parameters: (A) root length, (B) root FW, (C) root DW, (D) root surface area, (E) root volume and (F) root diameter. The comparisons among the treatments were made by applying post hoc Tukey's HSD test with small letters placed over the column indicating different treatment groups based on significant differences at *P* ≤ 0.05.

**Figure 11:**
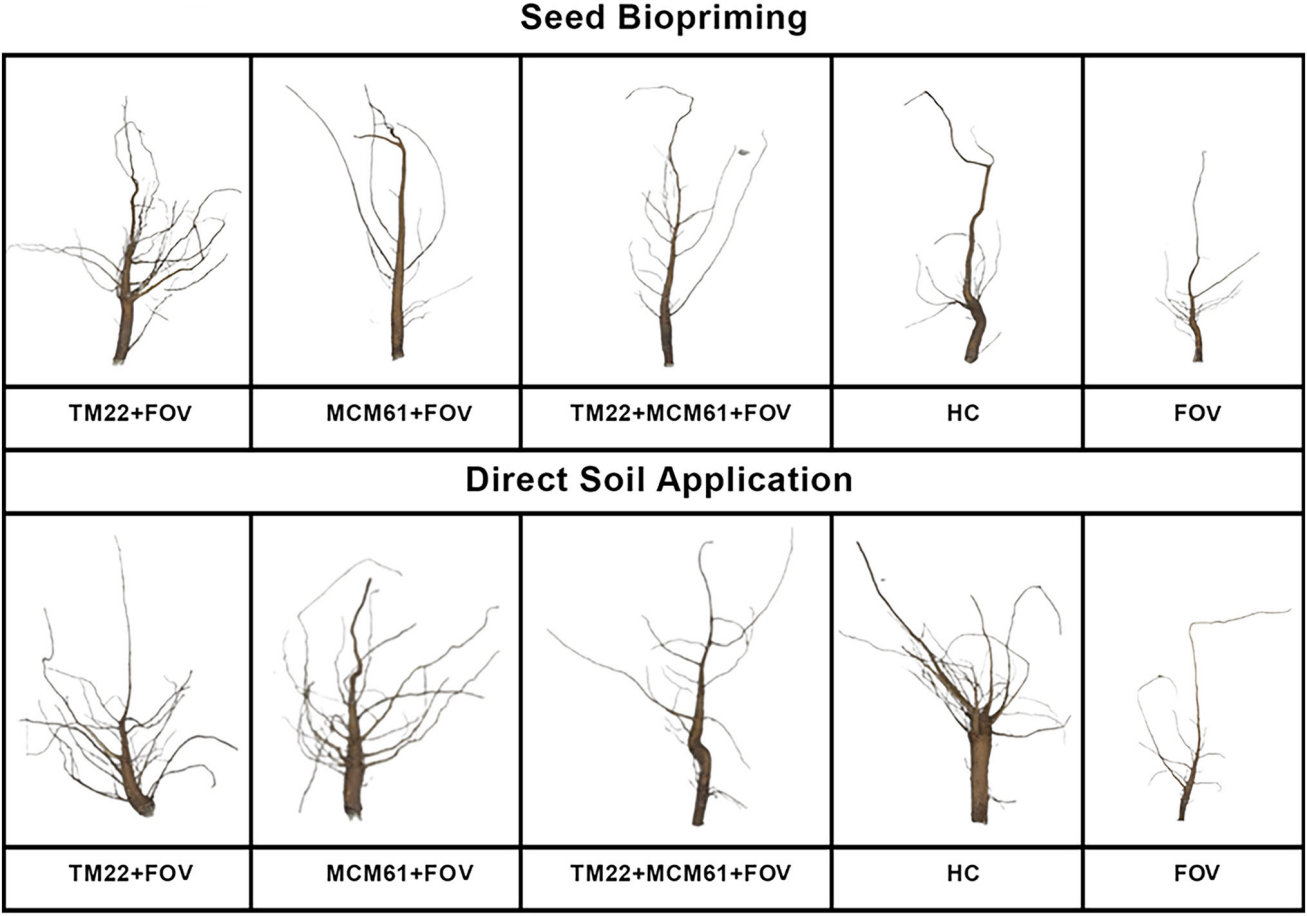
Rhizoscanning images of cotton plant roots subjected to treatment with *Bacillus* species applied via seed biopriming and soil drenching.

**Figure 12:**
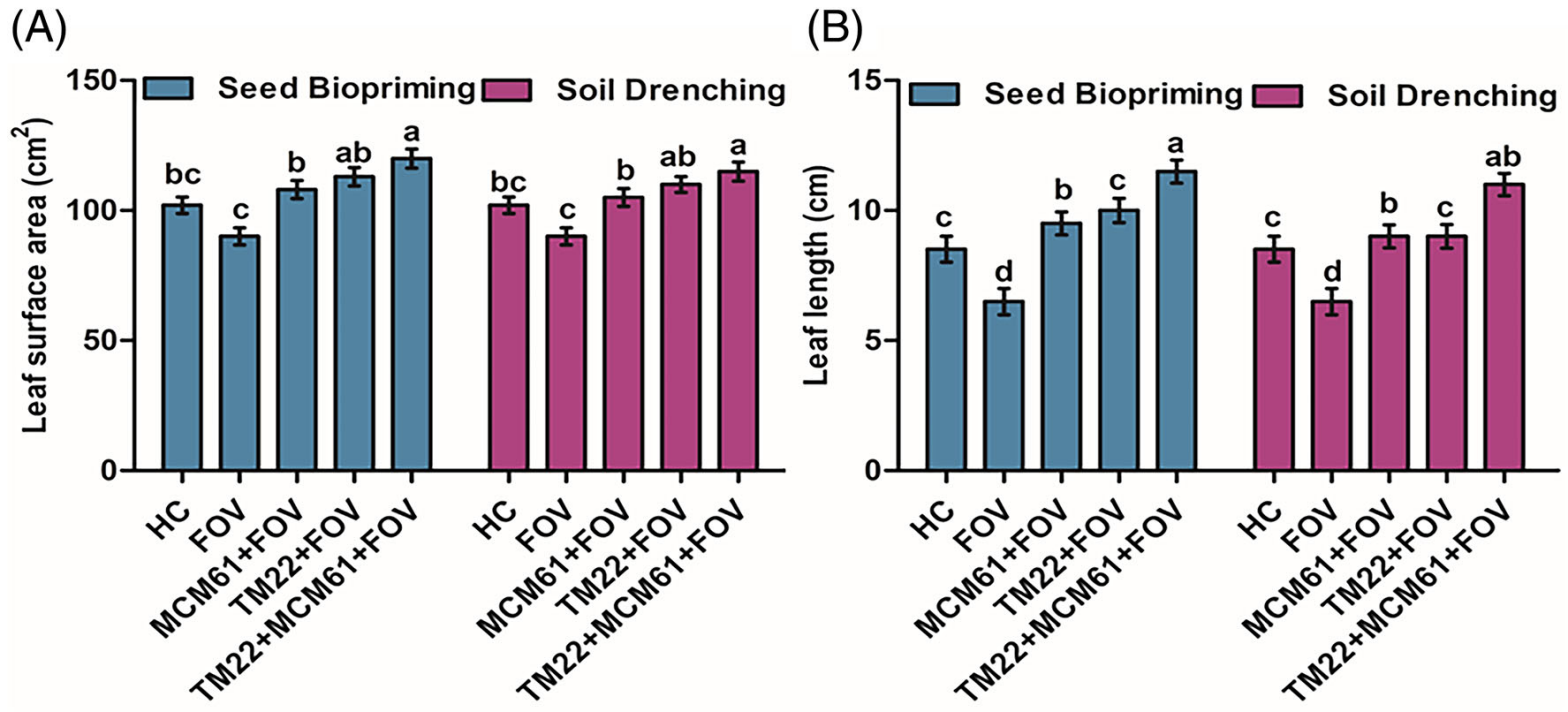
Effect of *Bacillus* species applied via seed biopriming and soil drenching on leaf‐growth parameters: (A) leaf surface area and (B) leaf length. The comparisons among the treatments were made by applying post hoc Tukey's HSD test with small letters placed over the column indicating different treatment groups based on significant differences at *P* ≤ 0.05.

The vegetative traits of cotton plants, including shoot length, shoot FW and shoot DW, were the highest in plants treated with co‐application of TM22 and MCM61 applied via seed biopriming compared to healthy control treatment [Fig. 9(A)–(C)]. The shoot length, shoot FW and shoot DW were the lowest in infected control plants, indicating that the pathogen infection negatively impacted the growth of the plants [Fig. 9(A)–(C)]. Likewise, the root parameters root length, root FW, root DW, root surface area, root volume and root diameter also were considerably high in plants treated with co‐application of TM22 and MCM61 and inoculated with FOV where the *Bacillus* species were applied as seed biopriming compared to soil drenching [Fig. 10(A)–(F)]. The images of rhizoscanning analysis of cotton plant roots subject to treatment with *Bacillus* species applied as seed biopriming and soil drenching are displayed in Fig. 11. The leaf scanning analysis revealed that leaf surface area and length also were the highest in plants treated with TM22 + MCM61 + FOV applied via seed biopriming compared to other treatments [Fig. 12(A), (B)]. Overall, seed biopriming with *Bacillus* species performed better than soil drenching application to improve the vegetative traits of cotton plants.

### 3.9 Effect of Bacillus species on physiological parameters

**Figure 13:**
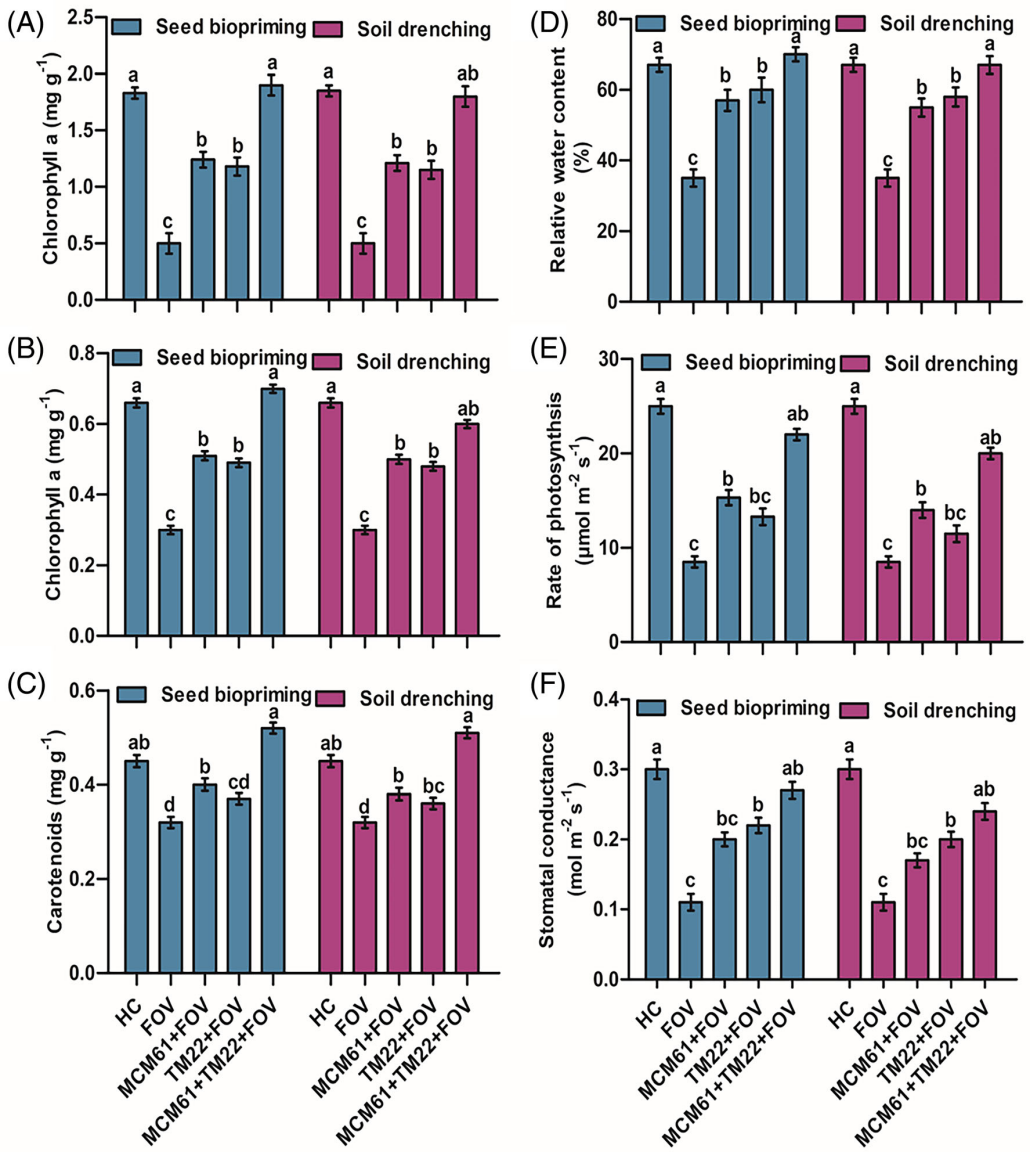
Effect of *Bacillus* species on physiological parameters of cotton plants: (A) chlorophyll a, (B) chlorophyll b, (C) carotenoids, (D) relative water content, (E) rate of photosynthesis and (F) stomatal conductance. The comparisons among the treatments were made by applying post hoc Tukey's HSD test, with small letters placed over the column indicating different treatment groups based on significant differences at *P* ≤ 0.05.

The treatment TM22 + MCM61 + FOV via seed biopriming showed the highest Chl a [Fig. 13(A)], Chl b [Fig. 13(B)], carotenoid contents [Fig. 13(C)], RWC [Fig. 13(D)], rate of photosynthesis [Fig. 13(E)], and stomatal conductance [Fig. 13(F)] compared to infected control (FOV), and healthy control treatment. Individual applications of TM22 and MCM61 also enhanced the Chl a, b, carotenoid content, RWC, rate of photosynthesis, and stomatal conductance compared to control treatments [Fig. 13(A)–(F)]. The lowest amount of Chl a, b, and carotenoid content, RWC, rate of photosynthesis, and stomatal conductance were observed in infected control plants [Fig. 13(A)–(F)].

### 3.10 Determination of stress markers

**Figure 14:**
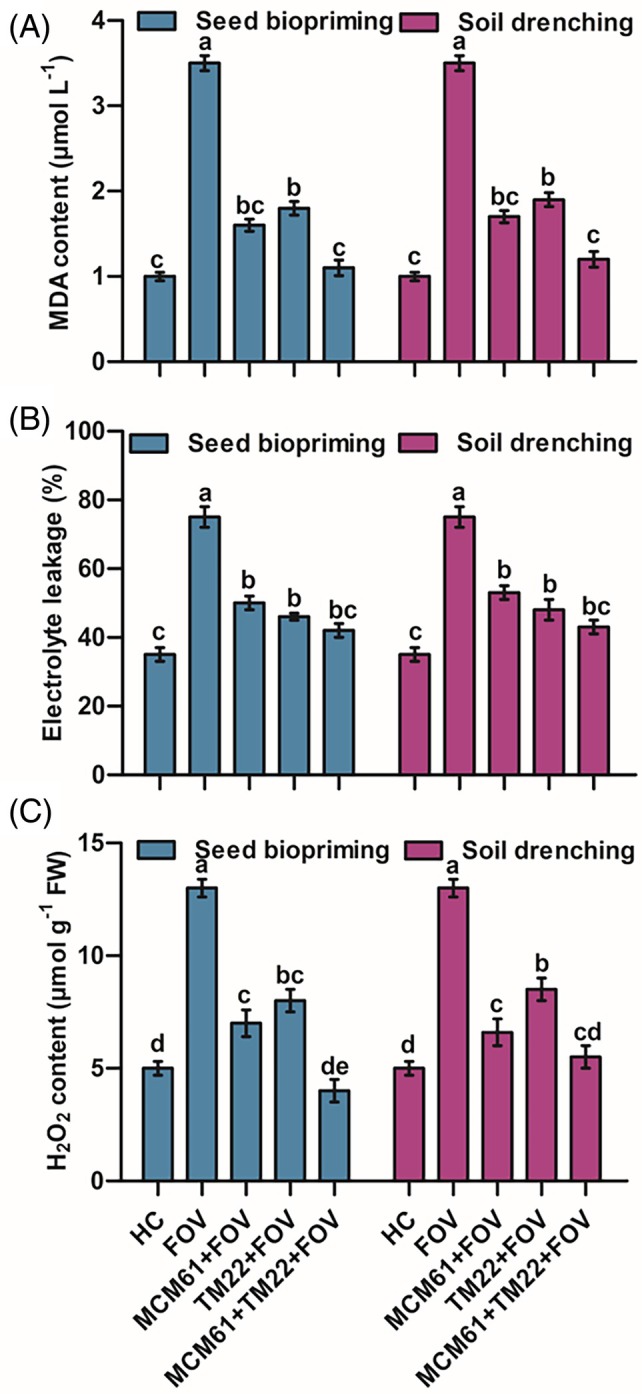
Effect of *Bacillus* species on oxidative stress markers: (A) MDA, (B) EL and (C) H_2_O_2_ contents. The comparisons among the treatments were made by applying post hoc Tukey's HSD test with small letters placed over the column indicating different treatment groups based on significant differences at *P* ≤ 0.05.

The activities of oxidative stress markers MDA, EL and H_2_O_2_ were the highest in infected control plants, indicating a high‐stress level in response to pathogen attacks. The treatment MCM61 + TM22 + FOV showed the lowest activities of MDA, EL and H_2_O_2_ [Fig. 14(A)–(C)]. Likewise, single applications of TM22 and MCM61 also reduced MDA, EL and H_2_O_2_ levels compared to infected control treatment [Fig. 14(A)–(C)].

### 3.11 Assessment of defense‐related enzyme activities

**Figure 15:**
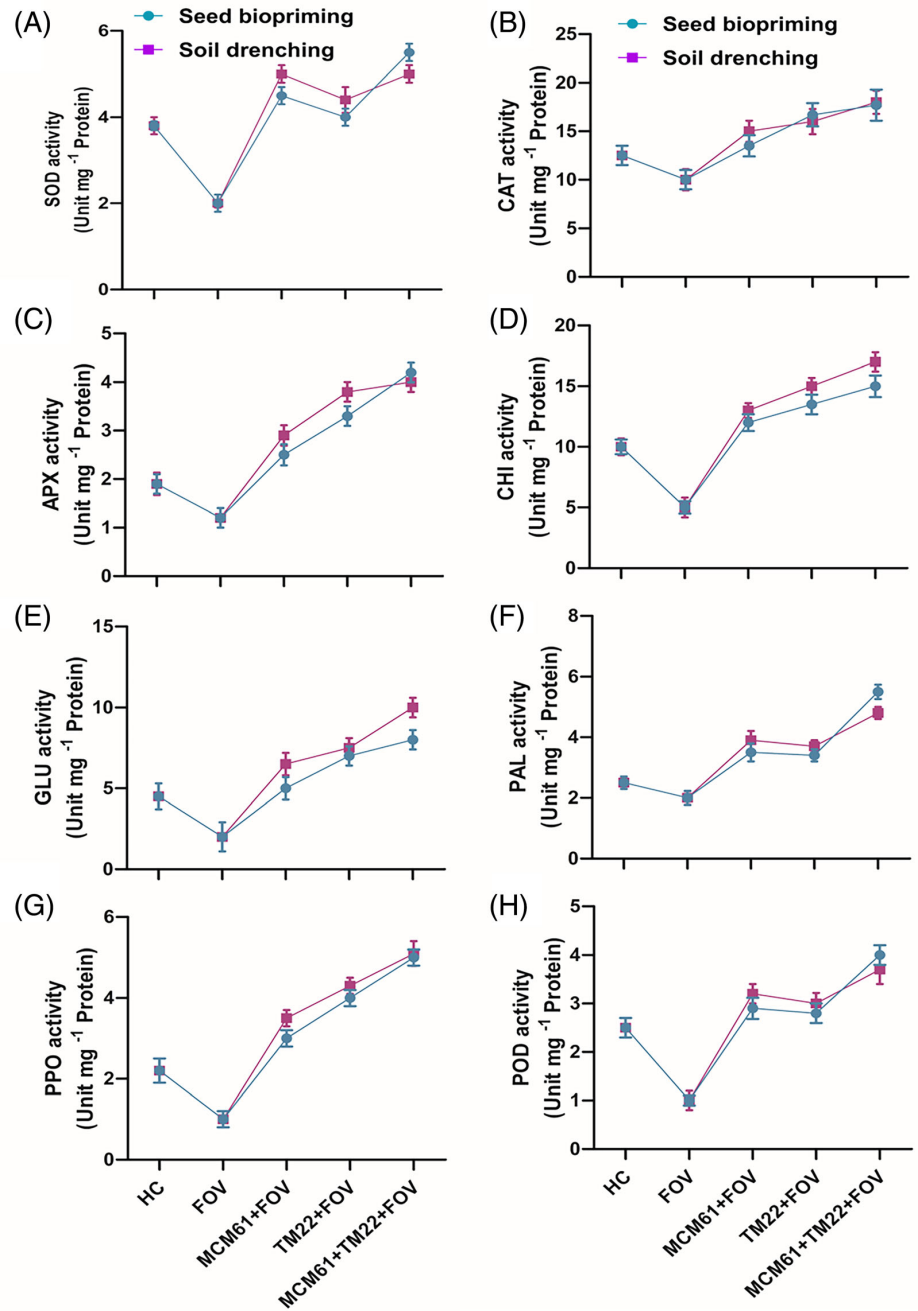
Effect of *Bacillus* species on the activities of defense enzymes of cotton plants: (A) SOD, (B) CAT, (C) APX, (D) CHI, (E) GLU, (F) PAL; (G) PPO and (H) POD. The comparisons among the treatments were made by applying post hoc Tukey's HSD test at *P* ≤ 0.05.

*Bacillus* species applied individually or in combination significantly enhanced the activities of all tested defense‐related enzymes compared to healthy control (HC) and infected control (FOV). The combined treatment TM22 + MCM61 + FOV showed the highest increase in all enzymes including SOD [Fig. 15(A)], CAT [Fig. 15(B)], APX [Fig. 15(C)], CHI [Fig. 15(D)], GLU [Fig. 15(E)], PAL [Fig. 15(F)], PPO [Fig. 15(G)] and POD [Fig. 15(H)] in both types of treatment applications; seed biopriming and soil drenching. However, a slightly higher increase in CHI, GLU, CAT and PPO was observed in soil drenching compared to seed biopriming, where POD, SOD, PAL and APX activities were higher in cotton plants treated with TM22 + MCM61 + FOV. The lowest activities of all tested enzymes were observed in the infected control treatment. Individual applications of TM22 and MCM61 under pathogen stress also enhanced the activities of POD, CAT, SOD, APX, GLU, CHI, PAL and PPO applied via seed biopriming and soil drenching compared to infected control and healthy control treatments.

### 3.12 Gene expression analysis

**Figure 16:**
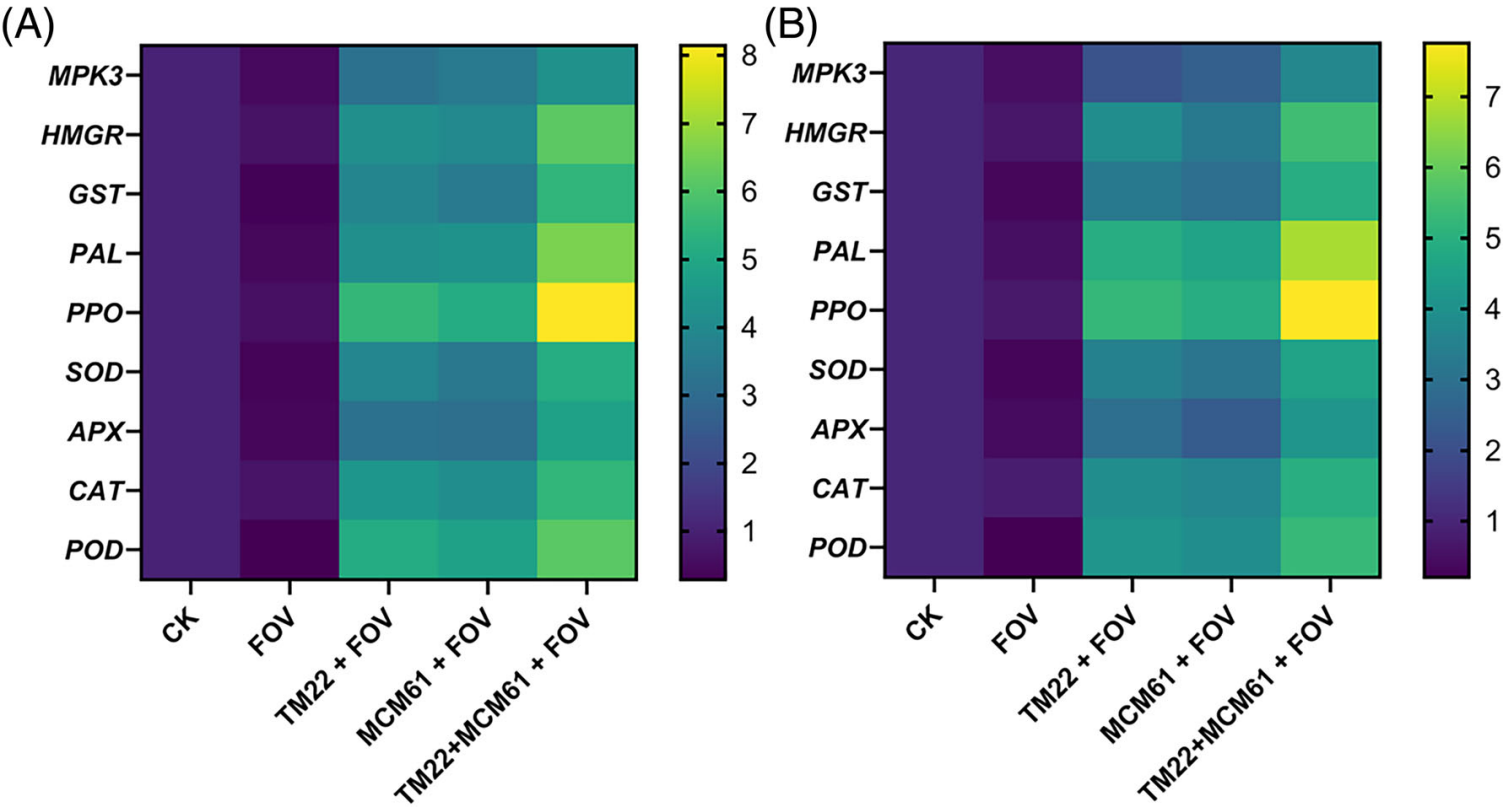
Gene expression analysis of defense‐related genes of cotton plants subjected to: (A) seed biopriming with *Bacillus* species and (B) soil drenching with *Bacillus* species.

The expression of defense‐related genes, including *MPK3, HMGR, GST, PAL, PPO, SOD, APX, CAT* and *POD* [Fig. 16(A), (B)], was the highest in cotton plants treated with co‐application of TM22 + MCM61 and inoculated with FOV compared to infected control and healthy control plants. The most highly upregulated genes were *PPO* (8.14‐fold), *PAL* (6.58‐fold), *HMGR* (6.14‐fold) and *POD* (6.13‐fold) in cotton plants treated with TM22 + MCM61 + FOV applied via seed biopriming, whereas in case of soil drenching the expression was *PPO* (7.75‐fold), *PAL* (6.78‐fold), *HMGR* (5.43‐fold) and *POD* (5.26‐fold). The expression of all defense‐related genes was slightly higher in plants subjected to seed biopriming compared to soil drenching. The lowest expression of all defense‐related genes was observed in infected control (FOV) treatment.

## 4 DISCUSSION

The rhizospheric soil serves as a primary reservoir of PGPR while providing both an environmentally friendly and sustainable approach for the effective management of plant diseases.^42^ The successful implementation of biological control methods requires the selection of new and novel antagonistic microorganisms with strong disease suppression effects. In this study, we identified two *Bacillus* species from cotton plant rhizosphere soil, including *B. altitudinis* TM22 and *B. atrophaeus* MCM61, because they showed great potential for antagonism of FOV, causing *Fusarium* wilt in cotton and promoted plant growth. *Bacillus* species reduce the soil pH and improve soil nutrient levels. They provide additional benefits to the plants by improving nutrient absorption and plant nutrient uptake.^43^

Endophytic *Bacillus* species adopt multifaceted modes to control plant pathogens and enhance plant growth.^44^ Among these MoAs, the synthesis of antimicrobial compounds, including LPs, PKs, hydrolytic enzymes and volatile compounds, is the most promising mechanism. In this study, *B. altitudinis* TM22 and *B. atrophaeus* MCM61 and their extracted LPs and PKs showed the most promising inhibition of FOV among the other *Bacillus* species in an *in vitro* plate assay. The *in vitro* inhibitory activity of *Bacillus* species is attributed to the production of antimicrobial compounds.^13^ *Bacillus* are considered antimicrobial compound‐producing factories because they produce a plethora of secondary metabolites with high antimicrobial activity.^45^ In support of our work, several previous research reports have documented the *in vitro* inhibitory activity of *Bacillus* strains against several fungal pathogens including *F. oxysporum*,^46^ *Verticillium dahliae*,^17^ *F. oxysporum* f. sp. *ciceris*,^14^ *F. solani* f. sp. *phaseoli*,^47^ *Penicillium italicum*,^48^ *Alternaria alternata*,^49^ *Magnaporthe oryzae*,^50^ and *P. digitatum*.^51^ The *in vitro* antagonism assays provide a baseline for screening the best *Bacillus* strains that can be used for field applications. The main cause of pathogen inhibition during *in vitro* assays stems from two factors: the competition between antagonist and pathogen, and the production of antimicrobial compounds.^52^ The bacteria produce antimicrobial compounds which cause inhibition of fungal pathogens during *in vitro* assays, which is visible by the production of inhibition zones around the fungal colonies.^53^ LPs and PKs are the key antimicrobial compounds responsible for causing the direct suppression of fungal pathogens.^54^ In this study, we have identified six LPs, including fengycin, iturin, surfactin, bacilysin, bacillibactin and bacillomycin, and one PK bacillaene using LC‐TOF/MS from *B. altitudinins* TM22. At the same time, only iturin was not detected in *B. atrophaeus* MCM61. *B. altitudinis* TM22 co‐produced three prominent families of LPs, including surfactin, fengycin and iturin in support of our previous work; *Bacillus* species co‐producing these three LPs were regarded as the most promising antagonists,^55^ and their additive efficacy has been documented against several plant pathogens.^56^, ^57^ Iturin is the principal inhibitory compound in the biological control activity of *Bacillus* species.^58^ Iturin makes pores in biological membranes, causing an osmotic disturbance.^59^ Iturin anchors into the lipid bilayer membrane and creates irreversible pores in the membrane by inserting surfactin molecules, which causes disruption and solubilization of the biological membrane.^60^ In agreement with our work, Kim *et al*.^61^ isolated a UV mutant strain BSM54 producing high levels of iturin with two‐fold higher inhibitory activity against *F. oxysporum* compared to wild‐type *B*. *velezensis*. They attributed the enhanced activity of BSM54 to the production of high levels of iturin. In previous studies, it has been reported that *Bacillus* strains co‐producing surfactin and iturin have higher antifungal activity, because surfactin makes mixed micelles with iturin.^62^, ^63^ Therefore, the higher inhibitory activity of *B. altitudinis* TM22 in our study can be attributed to the co‐production of surfactin and iturin. Surfactin has promising surface‐ and membrane‐active, emulsifying and foaming properties.^64^ *Bacillus* bacteria with surfactin‐producing ability exhibit biofilm formation and swarming motility, facilitating bacterial cell movement. Fengycin is another essential LP showing considerable inhibition of filamentous fungi.^52^ It interacts with the lipid bilayer membrane, leading to membrane solubilization, altered membrane structure and permeability, and the creation of ion channels in the lipid bilayer membrane.^65^ Likewise, the production of bacillomycin, bacilysin, bacillibactin and bacillaene also has been reported to be antimicrobial.^66^ The synergistic action of LPs and PKs has been reported in a previous study where mutant *Bacillus* strains deficit in any LP or PK biosynthesis have exhibited low antagonistic activity supporting the importance of synergism.^67^, ^68^ For instance, some LPs may not be strongly antimicrobial by themselves but they enhance the activity of other LPs. For example, surfactin facilitates biofilm formation and enhances membrane penetration of iturin. It has been reported that the antagonistic activity is significantly lower when certain LPs are missing in *Bacillus*. For example, a double mutant of *B. amyloliquefaciens* FZB42 lacking both bacillomycin and fengycin showed no suppression of *F. graminearum*.^69^ Therefore, the co‐production of these three LPs and other LPs and PKs by *Bacillus* species can be considered the cause of the promising antifungal potential of *B. altitudinis* TM22.

*Bacillus altitudinis* TM22 exhibited a strong ability to produce HCN in a qualitative test where the filter paper turned reddish‐brown. HCN‐producing *Bacillus* species are safe for the plants with no reported detrimental effects on plant health.^70^ HCN is a systemic poison, and its toxic effect is caused by its ability to inhibit crucial enzymes such as cytochrome c oxidase, which is responsible for causing suppression of *Fusarium* species.^71^ HCN is a potent secondary metabolite which disrupts cellular respiration and prevents oxygen‐use efficiency in plant pathogens. It interferes with the electron transport chain by irreversible binding with iron‐containing cytochrome c oxidase (complex IV) in mitochondria of fungal cells and ultimately blocks the final step of aerobic respiration.^72^ It also inhibits the reduction of O_2_ to H_2_O, halts the process of ATP production resulting in loss of energy in fungal cells.^72^ In an earlier report by Agha *et al*.,^70^ *B*. *velezensis* showed the ability to produce HCN and suppress *F. oxysporum* in wheat plants (*Triticum aestivum* L.). Ahmad *et al*.^73^ reported in their study that 50% of the tested *Bacillus* isolates were able to produce HCN, which was correlated with their potential to suppress plant pathogens.

Furthermore, in this investigation, treatment of cotton seeds with *B. altitudinis* TM22 and *B. atrophaeus* MCM61 showed more pronounced suppression of *Fusarium* wilt and improved vegetative traits of cotton plants compared to soil drenching. Most previous studies have used only the direct application method in the soil. However, we compared the efficacy of two treatment delivery methods; seed biopriming and soil drenching, for their antagonistic effect against *Fusarium* wilt in cotton plants. Seed priming with *Bacillus* strains had a more positive effect on reducing the disease development and enhancing the plant biomass compared to soil drenching. In agreement with our study, Zhang *et al*.^74^ reported that seed priming of cotton seeds with *B. subtilis* and *Gliocladium viren*s reduced the infection of *Fusarium* wilt disease, resulting in more vigorous and higher‐yielding plants. In another instance, Hasan *et al*.^17^ reported a substantial decrease in the development of *Verticillium* wilt in *Bacillus*‐treated cotton plants. Likewise, Akram *et al*.^75^ reported that coating seeds with the *B. aryabhattai* strain Z‐48 suppressed *Fusarium* wilt disease in tomato plants and enhanced the biomass. In this study, we have compared the effects of seed biopriming and soil drenching with *Bacillus* application on the suppression of *Fusarium* wilt and growth promotion of cotton plants. However, there is a lack of research reports on the comparative effects of seed biopriming and soil drenching with *Bacillus* on the suppression of plant diseases and plant growth promotion. Seed coating with PGPR *Bacillus* species helps to protect seeds and seedlings against pathogen attack during seed germination and development while simultaneously promoting plant growth.^76^ Seed priming with *Bacillus* species enables the bacteria to colonize the seeds and protect the plants from disease through direct or indirect mechanisms, including competition for space and nutrient acquisition, production of antimicrobial compounds, hydrolytic enzymes and the induction of systemic resistance.^77^ By applying *Bacillus* directly to the seeds, it ensures better colonization of the soil, providing a competitive edge over plant pathogens during the early growth stages of plants.^76^ The close association between *Bacillus* and plants induces systemic defense, improves germination rate and seedling vigor, and also promotes biofilm formation, which ultimately results in better plant growth. Seed biopriming with *Bacillus* species also improves physiological processes in the seed and, thus, plant growth.^78^ Compared to seed biopriming, soil drenching results in poor survival of biological control agents as a consequence of competition, inefficient root colonization and environmental instability.^79^ In agreement with our findings, several previous studies have reported that seed biopriming with *Bacillus* performed consistently better to suppress plant diseases and improve plant growth compared to soil drenching.^76^, ^79^ In our study the growth parameters of cotton plants were significantly improved in *Bacillus*‐treated plants. Several previous studies have reported the growth‐promoting effect of endophytic *Bacillus* species on a variety of plants, including chilli pepper (*Capsicum annum* L.),^80^ tomato (*Solanum lycopersicum* L.),^81^ tobacco (*Nicotiana tabacum*),^82^ cotton^5^ and soybean.^83^ *Bacillus* species promote plant growth by producing growth hormones such as indole‐3‐acetic acid and gibberellic acid, and through phosphate solubilization and siderophore production.^5^, ^84^ In the control treatment herein LB broth was used which had no reported effects on plant growth and pathogen inhibition.

The use of *Bacillus* as a bio‐fungicide through seed biopriming and soil‐drenching application methods boosted carotenoid levels, together with chl a and chl b contents, alongside improved RWC, photosynthetic rate and stomatal conductance in plants under pathogen attack. A previous investigation by Xie *et al*.^85^ showed that *B. subtilis* OKB105 species enhances photosynthesis rate together with chlorophyll production in tobacco plants. Likewise, Rashid *et al*.^86^ reported that the biomass of wheat plants was increased after treatment with *B. megaterium*, and the amount of photosynthetic pigments, RWC and osmolytes was improved under drought conditions. Previous studies also have reported that the application of *Bacillus* species resulted in higher rates of photosynthesis‐related gene expression and improved photosynthetic performance.^87^, ^88^ The findings of our study indicate that applying *Bacillus* strains improved the physiological processes in cotton plants. The positive and growth‐promoting effects of *Bacillus* species are likely to be responsible for the increased plant biomass and enhanced rate of physiological processes.

This research showed that *Bacillus*‐treated cotton plants displayed reduced stress markers, including EL, H_2_O_2_ and MDA, whereas the infected control plants maintained high‐stress marker levels. In a previous study, the application of *B. subtilis* combined with *Trichoderma viride* reduced the levels of H_2_O_2_, EL and MDA, thus preventing *Rhizoctonia fragariae* from causing black root rot in strawberries.^89^ Under drought and heat stress conditions, *B. mycoides* strain A3 effectively decreased the H_2_O_2_, EL and MDA levels in *Arabidopsis thaliana* seedlings.^90^ The cell membrane is a dynamic structure involving several physiological and biochemical processes which control cell membrane permeability. Plant pathogens penetrate the cell membranes directly, rapidly changing membrane permeability.^91^ The pathogen attacks the plant cells directly, resulting in enhanced electrolyte leakage.^92^ MDA is the final product in the process of lipid peroxidation.^93^ Lipid peroxidation is another physiological process triggered by free radicals causing membrane damage. The earliest defense mechanism of plants in response to pathogen attack is oxidative burst where reactive oxygen species (ROS) are released.^94^ H_2_O_2_ is a ROS that is converted into O_2_ and H_2_O, and triggers lipid peroxidation and cellular damage. The high accumulation of ROS is lethal for cells because unpaired electrons scavenge the other electrons from cell molecules to acquire stability, resulting in protein oxidation, lipid peroxidation and cellular damage.^89^ Cellular damage results in increased electrolyte leakage from the cell, ultimately leading to cell death.^95^ Therefore, the decrease in EL, MDA and H_2_O_2_ levels can be attributed to the effect of *Bacillus* species, which has reduced cell damage and maintained cell membrane integrity.

In order to further understand the potential of *Bacillus* species in defense elicitation, the activity of defense‐related enzymes was evaluated in treated cotton plants. Both *Bacillus* species TM22 and MCM61 enhanced the activity of plant defense‐related enzymes and conferred enhanced plant defense. *Bacillus* species have been reported to improve plant defense activity against biotic and abiotic stresses by enhancing the activities of defense enzymes, including SOD, POD, CAT, PPO and PAL.^96^ In a previous study, Wu *et al*.^97^ reported that *B. subtilis* SL‐44 enhanced the activity of SOD, PPO, PAL, CAT and POD to protect pepper plants (*Capsicum annuum*) from *Rhizoctonia solani* infection. Another study by Chen *et al*.,^67^ supported our findings, where *B. subtilis* B579 increased the activity of POD, PAL and PPO against *Fusarium* wilt of cucumber. Xu *et al*.^98^ reported that *B. siamensis* N‐1 reduced the disease incidence of soft rot in pitaya caused by *F. oxysporum* by regulating the activity of GLU, CHI, PAL, PPO and POD enzymes. Each of these enzymes plays vital and unique roles in plant defense. The phenylpropanoid pathway is an essential plant metabolic process that produces several toxic phenols and flavonoids. POD is an important defense enzyme that takes part in the final stage of lignin biosynthesis and converts phenolic compounds into very toxic quinones.^49^

The enzyme PPO converts phenols and lignin into extremely harmful quinones, inhibiting pathogens.^51^ The biochemical function of CAT is to convert H_2_O_2_ into O_2_ gas and H_2_O, which function together as O‐scavenging components.^99^ Through the breakdown of superoxide anion (O^2•−^), SOD prevents cellular damage by generating harmless end‐products.^100^ The enzyme APX functions like SOD by breaking down H_2_O_2_ to O_2_ gas and H_2_O by using ascorbate as an electron donor for protecting plant cells from oxidative damage.^101^ The main structural component of fungal cell walls, chitin, is destroyed by the action of chitinase enzyme, which results in weakening or destruction of the cell wall of fungal pathogens.^102^ Therefore, it is concluded that these defense‐related enzymes utilize diverse MoAs to protect the plants from pathogen attack.

The expression of defense‐related genes *HMGR*, *GST*, *PAL*, *PPO*, *MPK3*, *APX*, *POD*, *CAT* and *SOD* was assessed in cotton plants in response to treatment with *Bacillus* species. The research findings by Chandrasekaran & Chun^103^ validated our study, which demonstrated that *SOD*, *CAT*, *PAL*, *PPO*, *POD* and *GLU* genes displayed enhanced activity in tomato plants treated with *B. subtilis* strain CBR05 against *Erwinia carotovora* subsp. *carotovora*. Hasan *et al*.^17^ studied how endophytic *Bacillus* species enhanced the expression of *MPK3*, *SOD*, *PAL*, *GST*, *PPO* and *HMGR* genes in upland cotton plants against *Verticillium* wilt disease. The use of *B. thuringiensis* reduced the infection of *Ralstonia solanacearum* in tomato plants by increasing the expression of defense‐related genes and provided protection against subsequent pathogen attacks.^104^ Plants are protected by the pathogen attack by increasing the expression of defense‐related genes and their transcription and translation into corresponding defense‐related compounds. The *MPK3* gene produces critical defense signaling proteins known as mitogen‐activated protein kinases which are involved in pathogen‐related signal transductions.^105^ The *MPK3* gene has been reported to be the key factor that offers initial resistance against *Botrytis cinerea* infection.^106^ The *GST* gene encodes the glutathione S‐transferase enzyme, an important detoxifying enzyme that enhances resistance against biotic and abiotic stresses.^107^ The HMG‐CoA reductase enzyme encoded by the *HMGR* gene catalyzes the first step in the mevalonate (MVA) pathway. Isoprenoids are synthesized in the MVA pathway, which is essential for defense elicitation against biotic and abiotic stresses.^108^, ^109^ Therefore, suppression of *Fusarium* wilt in cotton is likely to be associated with the potential of *Bacillus* species to enhance the activity of defense‐related enzymes and expression of relevant genes.

## 5 CONCLUSION

Conclusively, our study demonstrates the promising antagonistic potential of *B. altitudinis* TM22 and *B. atrophaeus* MCM61 to protect cotton plants from *Fusarium* wilt disease in a series of *in vitro* and glasshouse experiments. Application of these *Bacillus* strains via seed biopriming and soil drenching reduced disease development. Moreover, these strains improved the biomass of cotton plants by improving physiological processes, defense‐related enzyme activity, the expression of defense‐related genes and by lowering the oxidative stress. These findings reveal that *Bacillus* species hold great promise as biocontrol agents for developing sustainable and eco‐friendly management strategies to control the *Fusarium* wilt of cotton. In the future, novel *Bacillus* strains with high antagonistic potential should be explored, and their applications at the field level must be tested.

## FUNDING INFORMATION

This research was funded by Taif University, Taif, Saudi Arabia, Project no. (TU‐DSPP‐2024‐37).

## CONFLICT OF INTEREST

The authors disclose no potential conflicts of interest.

## AUTHOR CONTRIBUTIONS

Conceptualization: T.M., A.M., F.Z., E.F., T.I.; methodology: T.M., A.M., M.N.A.; software: O.M.A., F.H.; validation: A.M., F.Z., F.H., A.F.A., S.S.A, A.A., S.A.; investigation: A.M., F.Z., F.H., A.F.A., S.S.A, A.A., S.A., T.I., data curation: T.M., A.M., F.Z., A.F.A., S.S.A, A.A., writing—original draft preparation: A.M., T.M.; writing—review and editing: T.M., A.M., F.Z., M.N.A., E.F., T.I., F.H., A.F.A., S.S.A, A.A., S.S.A., A.A., S.A; supervision: A.M., M.N.A.; project administration: A.M.

## Supporting information


**Fig. S1.** Maximum‐likelihood phylogenetic tree of *F. oxysporum* f. sp. *vasinfectum*. The evolutionary distances are computed using the Tamura–Nei model. The gaps and missing data were completely eliminated resulting in a final set comprising 574 positions.
**Fig. S2.** Neighbor‐joining phylogenetic tree of *Bacillus* species. The optimal tree shown in this figure has a sum of branch length 1.751. The tree is drawn to scale where the evolutionary distances are computed using the Tamura–Nei model. All ambiguous positions were deleted using pairwise deletion method with a final set comprising 2478 positions.

## Data Availability

The data that support the findings of this study are available from the corresponding author upon reasonable request.
