# Supplementary material for: Comparative efficacy of seed biopriming and soil drenching with Bacillus altitudinis TM22 and Bacillus atrophaeus MCM61 on the suppression of Fusarium wilt of cotton
Source: Pest Manag Sci. 2025 Nov 17;82(3):2424–45. doi: 10.1002/ps.70380 (PMC12886165; doi:10.1002/ps.70380)
Supplement: Supplementary file 1 — Fig. S1. Maximum‐likelihood phylogenetic tree of F. oxysporum f. sp. vasinfectum. The evolutionary distances are computed using the Tamura–Nei model. The gaps and missing data were completely eliminated resulting in a final set comprising 574 positions. Fig. S2. Neighbor‐joining phylogenetic tree of Bacillus species. The optimal tree shown in this figure has a sum of branch length 1.751. The tree is drawn to scale where the evolutionary distances are computed using the Tamura–Nei model. All ambiguous positions were deleted using pairwise deletion method with a final set comprising 2478 positions. [file PS-82-2424-s001.docx]

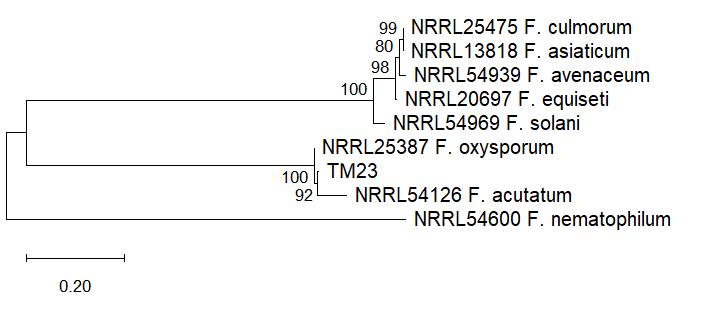


**Fig S1.** Maximum Likelihood phylogenetic tree of *F. oxysporum* f. sp. *vasinfectum*. The evolutionary distances are computed using Tamura-Nei model. The gaps and missing data were completely eliminated resulting in a final set comprising 574 positions.


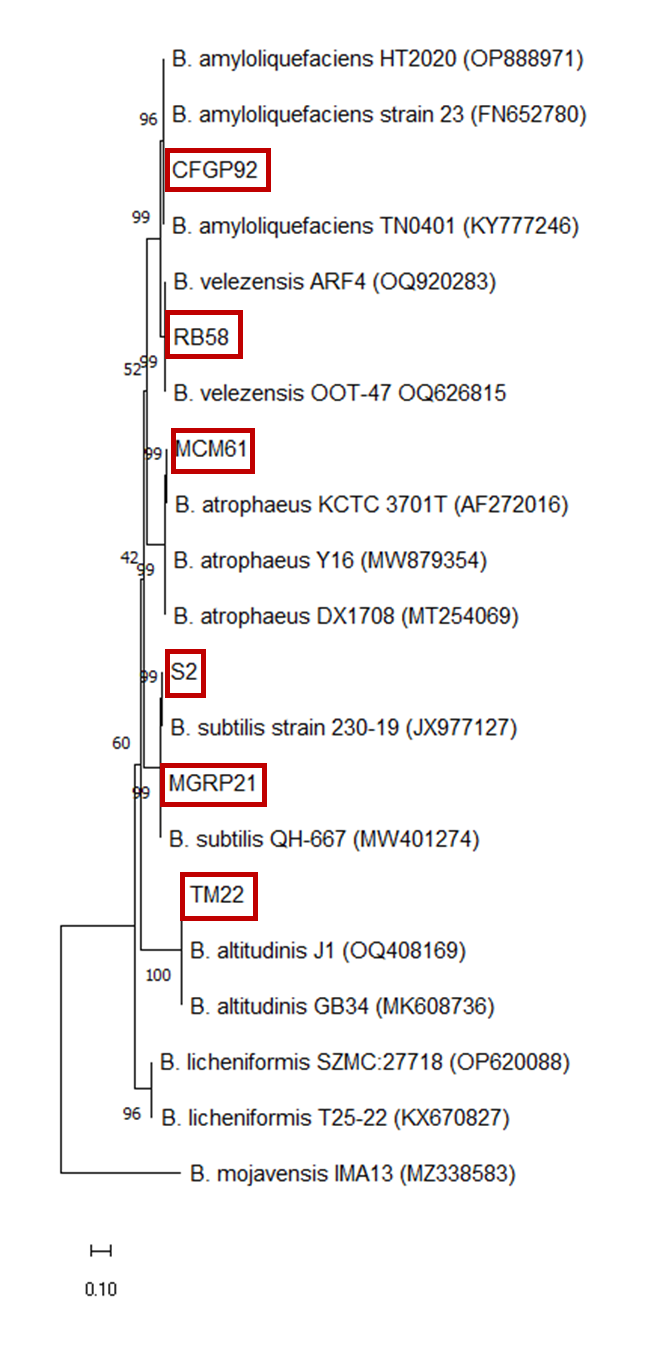


**Fig S2.** Neighbor joining phylogenetic tree of *Bacillus* species. The optimal tree shown in this figure has a sum of branch length 1.751. The tree is drawn to scale where the evolutionary distances are computed using Tamura-Nei model. All ambiguous positions were deleted using pairwise deletion method with a final set comprising 2478 positions.
